# Supplementary material for: Nutrition-Responsive Glia Control Exit of Neural Stem Cells from Quiescence
Source: Cell. 2010 Dec 23;143(7):1161–73. doi: 10.1016/j.cell.2010.12.007 (PMC3087489; doi:10.1016/j.cell.2010.12.007)
Supplement: Document S1. Article Plus Supplemental Information [file mmc1.pdf]

Cell

Nutrition-Responsive Glia Control Exit of Neural Stem Cells from Quiescence

James M. Chell[1] and Andrea H. Brand[1,*]
[1]The Gurdon Institute and Department of Physiology, Development, and Neuroscience, University of Cambridge, Tennis Court Road, Cambridge CB2 1QN, UK
*Correspondence: ahb@mole.bio.cam.ac.uk
DOI 10.1016/j.cell.2010.12.007

SUMMARY

The systemic regulation of stem cells ensures that they meet the needs of the organism during growth and in response to injury. A key point of regulation is the decision between quiescence and proliferation. During development, *Drosophila* neural stem cells (neuroblasts) transit through a period of quiescence separating distinct embryonic and postembryonic phases of proliferation. It is known that neuroblasts exit quiescence via a hitherto unknown pathway in response to a nutrition-dependent signal from the fat body. We have identified a population of glial cells that produce insulin/IGF-like peptides in response to nutrition, and we show that the insulin/IGF receptor pathway is necessary for neuroblasts to exit quiescence. The forced expression of insulin/IGF-like peptides in glia, or activation of PI3K/Akt signaling in neuroblasts, can drive neuroblast growth and proliferation in the absence of dietary protein and thus uncouple neuroblasts from systemic control.

INTRODUCTION

The stem cell populations found in tissues as varied as blood, gut, and brain spend much of their time in a mitotically dormant, quiescent state (for reviews, see Ma et al., 2009; Moore and Lemischka, 2006; Woodward et al., 2005; Zammit, 2008). Cellular quiescence, or G_0, is the reversible arrest of growth and proliferation and is actively maintained by a distinct transcriptional program (Coller et al., 2006). The balance between quiescence and proliferation, as well as the rate and duration of proliferation, can have significant effects on the growth, maintenance, and repair of tissues. When "choosing" whether or not to exit the quiescent state and divide, stem cells integrate a variety of local and systemic signals (reviewed in Drummond-Barbosa, 2008; Morrison and Spradling, 2008). In the mammalian brain, the neural stem cells (NSCs) in the subventricular zone (SVZ) and hippocampal subgranular zone (SGZ) transition between quiescence and proliferation, generating new neurons throughout the life of the animal (Ahn and Joyner, 2005; Doetsch et al., 1999; Ma et al., 2009; Morshead et al., 1994). A number of factors have been shown to have mitogenic effects on NSCs; however, it is not clear upon which cells (stem cells or their proliferative progeny) and at what point in the cell cycle these factors act (Zhao et al., 2008).

Drosophila neural stem cells (neuroblasts) in the central brain and thoracic ventral nerve cord (tVNC) are quiescent for ~24 hours between their embryonic and larval phases of proliferation (Hartenstein et al., 1987; Ito and Hotta, 1992; Prokop and Technau, 1991; Truman and Bate, 1988). Quiescent neuroblasts are easily identifiable and are amenable to genetic manipulation, making them a potentially powerful model with which to study the transition between quiescence and proliferation. However, the mechanisms regulating the exit from quiescence, either intrinsic or extrinsic, are not well established. Genetic studies found that *Drosophila* FGF, in concert with *Drosophila* Perlecan, promotes the neuroblast transition from quiescence to proliferation (Park et al., 2003), but subsequent work revealed that this effect is indirect (Barrett et al., 2008). Britton and Edgar found that the exit from quiescence is physiologically coupled to larval growth and development via a nutritional stimulus (Britton and Edgar, 1998). The *Drosophila* fat body performs many of the storage and endocrine functions of the vertebrate liver and acts as a sensor, coupling nutritional state to organismal growth (Colombani et al., 2003). In response to dietary amino acids, the fat body secretes a mitogen that acts on the CNS to bring about neuroblast proliferation (Britton and Edgar, 1998). This fat body-derived mitogen (FBDM) initiates cell growth in quiescent neuroblasts and promotes (or at least permits) cell-cycle re-entry (Britton and Edgar, 1998). Yet the identity of the FBDM, the cell type upon which it acts, and the downstream pathway activated in neuroblasts are unknown.

Insulin and insulin-like growth factor (IGF) signaling are powerful regulators of growth and metabolism. In mammals, IGF-I has been shown to drive the proliferation of neural stem cells in both the embryo and adult (reviewed in Anderson et al., 2002; Joseph D'Ercole and Ye, 2008). IGF-I expression is induced in astrocytes (astroglia) in response to a variety of CNS injuries (Yan et al., 2006; Ye et al., 2004) and is thought to be responsible for the increased neural stem cell proliferation seen in the SVZ and SGZ following cortical ischemia (Yan et al., 2006).

In *Drosophila*, there are seven insulin/IGF-like peptides (dILPs 1–7) and a single insulin/IGF receptor (dInR). dInR activates the highly conserved PI3K/Akt pathway, leading to cellular growth and proliferation (reviewed in Goberdhan and Wilson, 2003). dILPs expressed by the IPC (insulin-producing cell) neurons of the brain are secreted into the circulation, where their endocrine

functions include the regulation of growth, carbohydrate metabolism, and germline stem cell division (Ikeya et al., 2002; LaFever and Drummond-Barbosa, 2005; Rulifson et al., 2002). dInR is strongly enriched in the developing CNS and its resident neuroblasts (Fernandez et al., 1995; Garofalo and Rosen, 1988), but a role for the insulin/IGF pathway in neuroblast proliferation has not been found.

We show here that the nutritional stimulus (known to be transduced by the fat body [Britton and Edgar, 1998]) induces the expression of dILPs in a subset of glia that neighbors neuroblasts and that the InR/PI3K pathway is required by neuroblasts for the exit from quiescence. Indeed, the forced expression of dILPs in glia, or activation of PI3K/Akt signaling in neuroblasts, can drive neuroblast proliferation in the absence of dietary protein, uncoupling the quiescence and proliferation of neuroblasts from systemic nutritional control. Thus, we identify a paracrine function of dILPs as mediators of the systemic regulation of neuroblast proliferation.

RESULTS

Neuroblast Reactivation and Nutritional Dependence

During embryogenesis, neuroblasts proliferate to generate the neurons that will form the larval CNS. Following the embryonic phase of proliferation, neuroblasts either enter into quiescence or undergo apoptosis. Quiescent neuroblasts reactivate and resume proliferation during larval stages, generating neurons that will contribute to the adult CNS (reviewed in Egger et al., 2008).

Neuroblasts exit quiescence during the first and second larval instars (~0–24 and 24–48 hr posthatching [hph], respectively) (Ito and Hotta, 1992; Truman and Bate, 1988). We have focused on the neuroblasts of the thoracic VNC (tVNC) (Figure 1 and Figure S2 available online), which have been thoroughly characterized during this period of development (Truman and Bate, 1988). In order to label and manipulate neuroblasts during the transition from quiescence to proliferation (reactivation), we generated a GAL4 line using a neuroblast-specific *grainyhead* enhancer (Prokop et al., 1998; Uv et al., 1997) (grh-GAL4). grh-GAL4 drives expression of UAS-linked genes in a subset of neuroblasts during reactivation (Figures 1A–1C). In combination with the neuroblast marker Deadpan (Dpn) (Bier et al., 1992), grh-GAL4 allows us to unequivocally identify, manipulate, and assay neuroblasts throughout reactivation.

At the beginning of the first larval instar, the cell body diameter of quiescent neuroblasts is ~3–4 μm, similar to surrounding neurons. Shortly thereafter, neuroblasts begin to enlarge, and by 24 hph, the average diameter is ~7 μm (compare Figures 1A and 1B). It is at this time that the first neuroblast divisions are seen (Truman and Bate, 1988 and data not shown). Neuroblasts reactivate asynchronously, but by the end of the second larval instar, all neuroblasts have fully enlarged and begun to proliferate (Truman and Bate, 1988; Figure 1C). Interestingly, the exit from quiescence of neural stem cells from the developing mammalian cortex has also been shown to coincide with an increase in cell size (Alam et al., 2004; Groszer et al., 2006).

Quiescent neuroblasts, like quiescent neural stem cells of the mammalian SVZ and SGZ, exhibit a more complex morphology than proliferating cells (Figure 1B′) (Ma et al., 2009). Quiescent neuroblasts extend a primary cellular process toward the neuropil and also occasionally extend a process toward the ventral surface or toward other neuroblasts (Truman and Bate, 1988; Figures 1A–1B′). These processes are present until neuroblasts begin to divide (Tsuji et al., 2008), but their function has not yet been investigated. In larvae, growth and cell proliferation are triggered by feeding (Britton and Edgar, 1998). In larvae reared on a sucrose-only (amino acid-deprived) diet, neuroblast reactivation never occurs. Neuroblasts display no cellular growth (a prerequisite for neuroblast cell cycle re-entry) and maintain their primary process (Britton and Edgar, 1998; Figures 1D–1F).

Stellate Surface Glia Express dILP6 and dILP2 during Reactivation

A transcriptome analysis comparing VNCs from newly hatched larvae and VNCs from larvae at the end of the first instar suggested that the expression of dILP6 and dILP2 increases in the VNC during neuroblast reactivation (J.M.C. and A.H.B., unpublished data). The seven dILPs are expressed in distinct spatiotemporal patterns during development (Brogiolo et al., 2001). dILP6 is reported to be expressed in the larval gut (Brogiolo et al., 2001) and the pupal fat body (Okamoto et al., 2009; Slaidina et al., 2009), whereas dILP2 is known to be expressed in the IPC neurons of the brain (along with dilps 1, 3, and 5) (Ikeya et al., 2002; Rulifson et al., 2002).

To determine whether dILP6 is also expressed in the CNS, we generated a dilp6-GAL4 line (see Experimental Procedures). dilp6-GAL4 drives expression in a subset of the surface glia that wraps the CNS (Figures 2A–2B′). Strong expression was evident by mid first instar (11 hph) and was maintained throughout neuroblast reactivation (Figures 2A–2B′). We also assayed the expression of dILP2 by immunohistochemistry and found that it too was expressed in the same surface glial population (Figures 2C and 2C′ and Figure S1). The glial cells labeled by dilp6-GAL4 are located above the neuroblasts and underneath the surrounding basement membrane (Figures 2D and 2E). They are stellate in appearance, with several processes radiating from the central cell body (Figures 2A–B′). Thus, dILPs, expressed by glial cells, are ideally positioned to activate the dInR pathway in neuroblasts during reactivation.

PI3K Is Active during, and Required for, Neuroblast Reactivation

dInR regulates growth and proliferation in other tissues by recruiting PI3K to the cell membrane, where it converts phosphoinositol(4,5)P2 (PIP2) to phosphoinositol(3,4,5)P3 (PIP3) (Leevers et al., 1996; Oldham et al., 2002; Weinkove et al., 1999). PIP3 then recruits the protein kinase Akt (among other proteins) to the membrane, leading to Akt activation and signaling (Stocker et al., 2002; Verdu et al., 1999). PI3K activity can be assayed with a pleckstrin homology (PH) domain-green fluorescent protein (GFP) fusion protein (PH-GFP) (Britton et al., 2002). PH-GFP is strongly recruited to the membrane when PIP3 levels are high (i.e., when PI3K is active) via the binding of its PH domain to PIP3. We observe a strong increase of membranous PH-GFP in reactivating neuroblasts (compare Figures S2A and S2A′ with S2B and S2B′), consistent with an

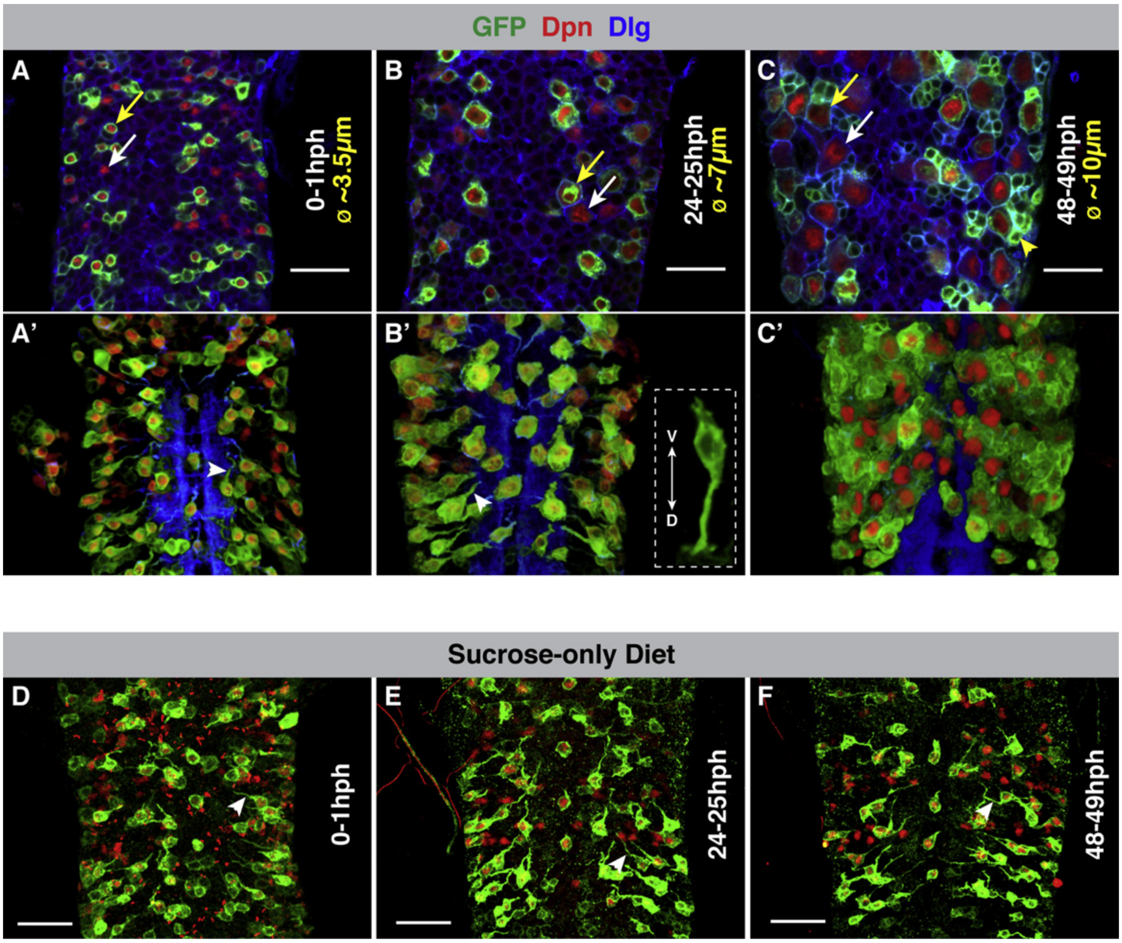

Figure 1. Nutritional Dependence of Neuroblast Reactivation

(A–F) grh-GAL4 drives strong expression of UAS-mCD8-GFP in one-third of neuroblasts in the thoracic VNC (tVNC) (~16/47 per thoracic segment; ~48/141 total). Yellow arrows highlight examples of grh-GAL4-expressing neuroblasts. White arrows highlight examples of neuroblasts that do not express grh-GAL4.

(A and A′) In just-hatched larvae (0–1 hours posthatching [hph]), the cell body diameter (ø) of a neuroblast is ~3–4 μm.

(B and B′) By 24 hph, most neuroblasts have increased in diameter but maintain their primary process (white arrowheads) prior to division. The dashed box in (B) shows a snapshot from a 3D reconstruction of a neuroblast (ventral, V; dorsal, D).

(C and C′) By 48 hph, neuroblasts have fully enlarged and undergone several divisions. Note the small GFP-marked, Dpn-negative progeny (e.g., yellow arrowhead).

(A′), (B′), and (C′) are snapshots from 3D reconstructions of the VNCs shown in (A), (B), and (C), respectively.

(D–F) In larvae deprived of amino acids (sucrose-only diet), neuroblast growth and cell-cycle re-entry never occur (Britton and Edgar, 1998). Neuroblasts maintain their quiescent size and primary process. Compare (D), (E), and (F) with (A′), (B′), and (C′), respectively. Z projections of tVNCs at indicated time points. GFP, green; Deadpan (Dpn; neuroblast nuclei, red); Discs Large (Dlg; cell cortices, blue). Scale bars, 20 μm.

increase in PI3K activity. We also see strong expression of S6 kinase (S6K) in reactivating neuroblasts (Figure S3), a kinase known to promote growth downstream of insulin/PI3K signaling (Lizcano et al., 2003; Miron et al., 2003; Rintelen et al., 2001).

While dInR null mutants are embryonic lethal (Fernandez et al., 1995), PI3K null mutants survive through larval development (Weinkove et al., 1999). Null mutants of the catalytic subunit of PI3K, dp110, display normal growth until the third larval instar. In these mutant larvae, the imaginal discs are not discernible; however, the CNS was reported to appear normal (Weinkove et al., 1999). We examined dp110 mutants and found that the CNS is significantly reduced in size compared to wild-type larvae (Figure S2). Such a reduction in CNS size is indicative of reduced neuroblast proliferation. The neuroblasts in dp110 mutants are severely reduced in size, with the majority showing no sign of postembryonic growth or division (Figure S2 and data not shown). These results demonstrate that PI3K signaling is required in order for neuroblasts to reactivate.

Inhibition of dInR/PI3K Signaling Retards the Exit from Quiescence

The neuroblast phenotype seen in dp110 null mutant larvae could result from either an intrinsic requirement for PI3K signaling within neuroblasts or a requirement for PI3K in another cell or tissue type that affects neuroblast reactivation. In order to address whether dInR and PI3K are intrinsically required by

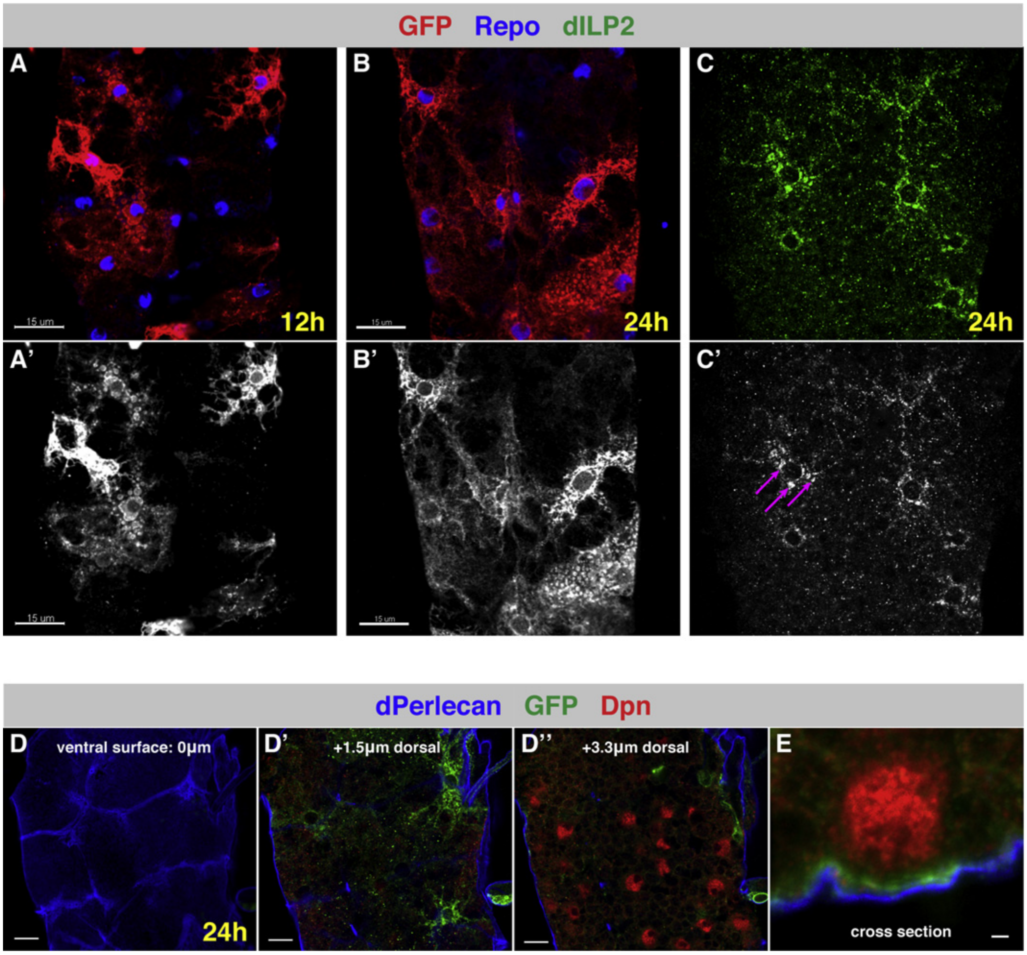

Figure 2. Glia Express dILP6 and dILP2 during Reactivation

(A–B′) dilp6-GAL4 marks a subset of the outermost, perineurial (Stork et al., 2008) glia during first- and second-larval instars. dilp6-GAL4-driving UAS-mCD8-GFP, red; glial nuclei, blue (anti-Repo). Scale bars, 15 μm.

(C) Anti-dILP2 (green) in the tVNC at 24 hph shows a punctate perinuclear enrichment in surface glial cells (see pink arrows in C′), consistent with secretory vesicle processing. Z projection of ventral surface glial layer.

(D and E) dILP6-positive glia (dilp6-GAL4 > UAS-mCD8-GFP [green]) lie just above neuroblasts (Dpn, red) and below the basement membrane (dPerlecan, blue). Sequential sections from ventral surface of VNC (D and D″) and in cross-section (E). Scale bars: D and D″, 10 μm; E, 1μm.

See also Figure S1.

neuroblasts for the exit from quiescence, we used grh-GAL4 to express negative regulators of the pathway within neuroblasts. By the end of the first larval instar (24 hph), the majority of neuroblasts in the tVNC have already enlarged significantly. The average neuroblast diameter increases from ~4 μm to ~7 μm (Figures 1A and 1B and Figures 3A and 3E). Expression of a dominant-negative form of the PI3K adaptor subunit (Δp60) (Weinkove et al., 1999) within neuroblasts caused a strong reduction in neuroblast growth during the first larval instar, with most neuroblasts maintaining their small quiescent size of ~4 μm (Figures 3A, 3B, and 3E). In *Drosophila*, as in vertebrates, the tumor suppressor PTEN antagonizes PI3K by converting PIP3 to PIP2 (Goberdhan et al., 1999; Maehama and Dixon, 1998). Misexpression of dPTEN (Huang et al., 1999) within neuroblasts generated the same phenotype as Δp60 expression, effectively blocking growth and reactivation during the first larval instar (Figures 3A, 3C, and 3E). These two results suggest that the PIP3-generating activity of PI3K is required intrinsically by neuroblasts for reactivation to occur. Finally, if dInR is responsible for activating PI3K, then blocking dInR function should phenocopy the expression of Δp60 or dPTEN. Expression of a dominant-negative form of dInR ($dInR^{K1409A}$) inhibits neuroblast reactivation in the same manner as Δp60 and dPTEN, with the majority of neuroblasts remaining ~4 μm in diameter (Figures 3A, 3D, and 3E). Neuroblasts that do not express grh-GAL4 act as an internal control, showing that neuroblast reactivation can occur as normal in these cells (see dashed boxes, Figures 3A–3D). These data support a model in which the activation of dInR in neuroblasts and the subsequent upregulation of PI3K are responsible for the exit from quiescence.

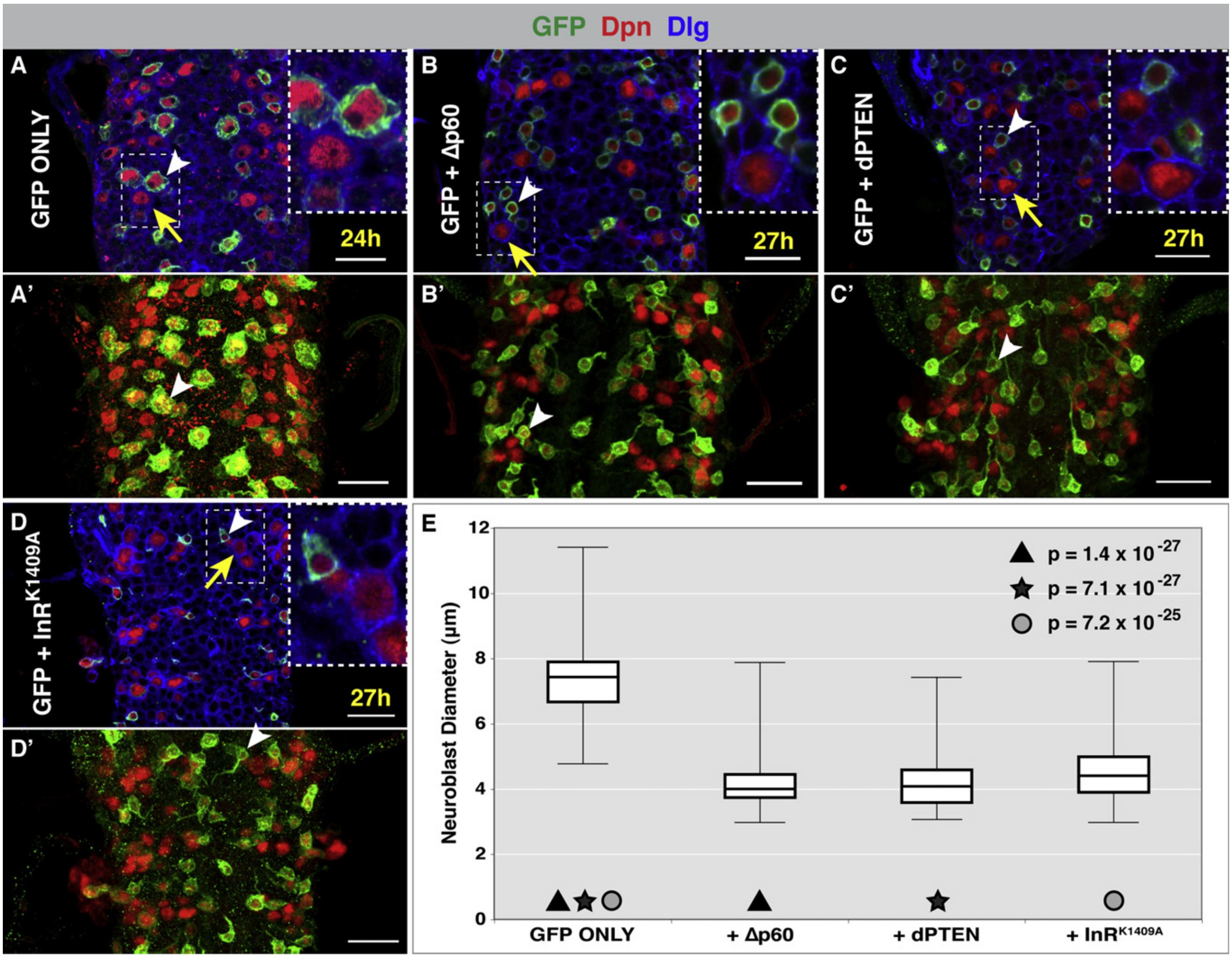

Figure 3. Neuroblast Reactivation Requires Cell-Intrinsic dInR/PI3K Signaling

grh-GAL4 driving: mCD8-GFP (A), mCD8-GFP + dominant-negative PI3K (Δp60) (B), mCD8-GFP + dPTEN (C), and mCD8-GFP + dominant-negative insulin receptor (dInR^{K1409A}) (D).

(A) By 24 hph, all neuroblasts in the tVNC have begun to enlarge, and average cell body diameter has increased from ~4 μm to ~7 μm.

(B–D) Expression of Δp60, dPTEN, or dInR^{K1409A} retards growth and cell-cycle re-entry (white arrowheads). Neuroblasts that do not express grh-GAL4 show normal cell growth (compare yellow arrows with white arrowheads). (A′–D′) are projections of VNCs shown in (A–D), respectively. White arrowheads in (A′–D′) point to the same neuroblasts as in (A–D), respectively.

(E) A quantification (box and whisker plot) of the experiments represented in (A–D). GFP only (control), n = 52 (6 VNCs), mean = 7.45 μm, SD = 1.24. +PI3K (Δp60), n = 62 (5 VNCs), mean = 4.21 μm, SD = 0.87. +dPTEN, n = 114 (12 VNCs), mean = 4.22 μm, SD = 0.76. +dInR^{K1409A}, n = 109 (12 VNCs), mean = 4.54 μm, SD = 0.94. (n equals number of neuroblasts assayed). p values were generated using Student's t test. GFP, green; Dpn, red; Dlg, blue. Scale bars, 20μm.

See also Figure S2 and Figure S3.

Activation of PI3K Is Sufficient for Neuroblast Reactivation

If the dInR/PI3K pathway is responsible for neuroblast reactivation in response to nutritional stimuli, then activation of the pathway in the absence of the stimulus might be expected to cause aberrant reactivation. In order to test this hypothesis, we expressed a membrane-targeted, constitutively active, version of the PI3K catalytic subunit (dp110^{CAAX}) (Leevers et al., 1996) in neuroblasts of larvae that were reared on a sucrose-only diet. We found that constitutive activation of PI3K can drive neuroblast reactivation during the first larval instar, irrespective of dietary protein (Figure 4A–4B′). High levels of PI3K activity increased the rate of reactivation beyond those normally seen; at the end of the first larval instar (24 hph), we find neuroblasts that have prematurely reached their full size (10 μm or more) and have already undergone multiple rounds of cell division (as evidenced by the presence of several small GFP-retaining daughter cells; Figure 4B). Thus, PI3K signaling within neuroblasts can drive the cellular growth and proliferation that constitute the exit from quiescence. The divisions proceed with the correct asymmetric partitioning of Miranda and Prospero into the differentiating daughter cell (reviewed in Knoblich (2008) (Figures 4C–4E).

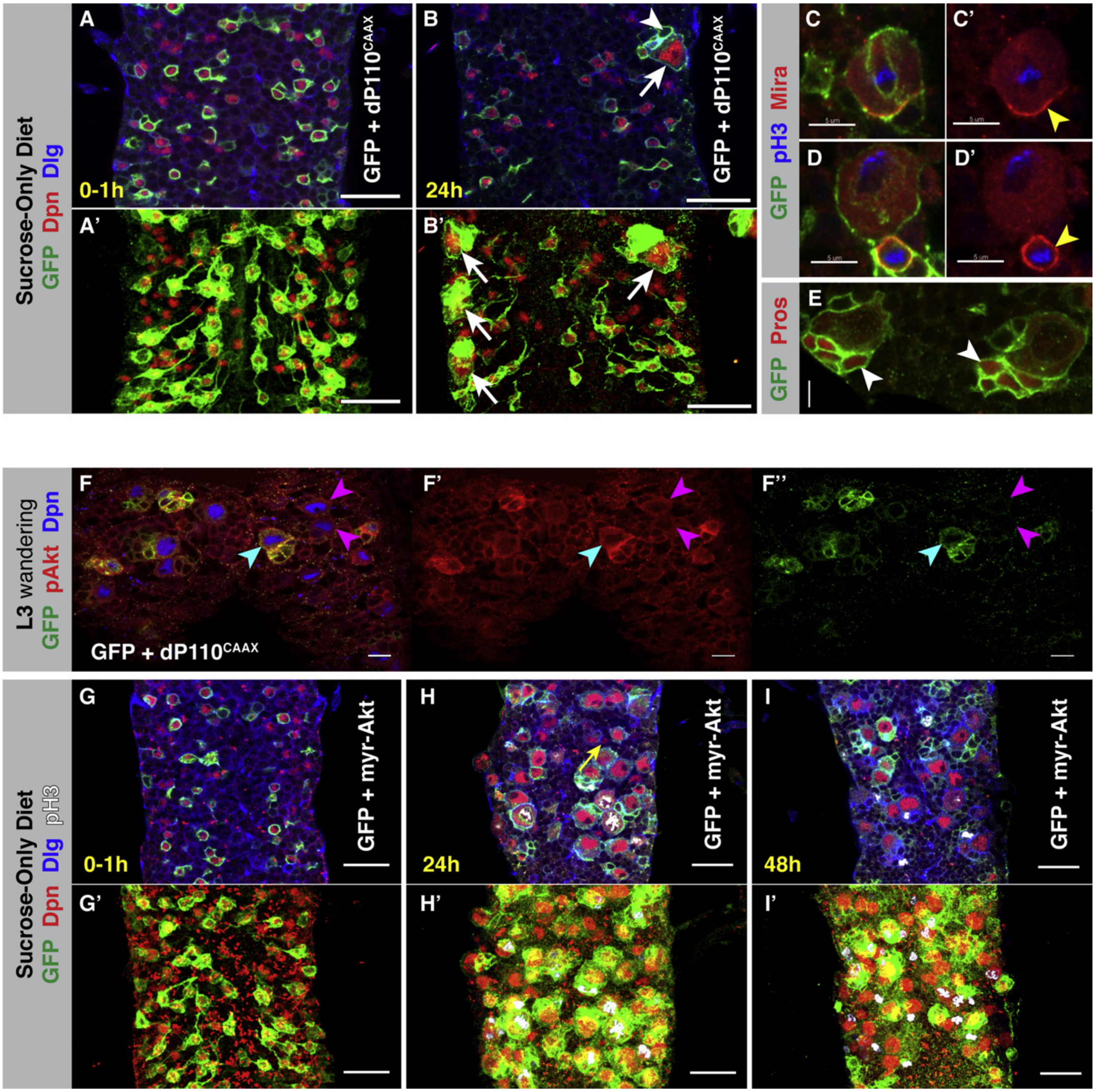

Figure 4. PI3K and Akt Signaling Are Sufficient for Neuroblast Reactivation

(A–E) grh-GAL4 driving UAS-mCD8::GFP (green) and UAS-dp110^{CAAX} (a constitutively active form of the PI3K catalytic subunit) in larvae fed a sucrose-only (amino acid-free) diet. (A′) and (B′) are projections of the VNCs in (A) and (B), respectively.

(A and A′) Neuroblasts in which PI3K signaling is activated by dp110^{CAAX} are quiescent at 0–1 hph. Scale bars, 20 μm.

(B and B′) Neuroblasts can fully reactivate during the first-larval instar despite the absence of a nutritional stimulus. Arrows in (B) and (B′) point to an enlarged, reactivated neuroblast. The arrowhead in (B) points to one of the progeny of a reactivated neuroblast. Dpn, red; GFP, green; Dlg, in blue. Scale bars, 20 μm.

(C and D′) The adaptor protein Miranda (red) is asymmetrically localized and partitioned to daughter cells of dp110^{CAAX}-reactivated neuroblasts (yellow arrowheads). Scale bar, 5 μm.

(E) The cell-fate determinant Prospero (red) is also partitioned to dp110^{CAAX}-reactivated neuroblast progeny (see white arrowheads). Scale bar, 5 μm.

(F–F″) Neuroblasts (Dpn, blue) in which PI3K signaling is upregulated by expression of dp110^{CAAX} show significantly increased levels of phosphorylated (active) Akt (pAkt, red) (blue arrowhead). Example control neuroblasts indicated by pink arrowheads. grh-GAL4 driving UAS-mCD8::GFP (green) and UAS-dp110^{CAAX} in third-instar larvae fed a normal diet (fresh yeast). Scale bars, 10 μm.

(G–I′) grh-GAL4 driving UAS-mCD8::GFP (green) and UAS-myr-Akt (a constitutively active form of Akt) in larvae fed a sucrose-only (amino acid-free) diet. (G′–I′) are projections of VNCs in (G–I), respectively. Dpn, red; Dlg, blue; pH3-labeled mitotic cells, white. Scale bars, 20 μm.

The activation of PI3K in this context appeared to cause reactivation in an all or nothing manner. We observed a subset of the grh-GAL4-positive neuroblasts reactivating fully. Of the 141 thoracic neuroblasts (Truman and Bate, 1988), ~48 show significant grh-GAL4 expression. Of these 48 neuroblasts, 2–6 (~4%–12%) reactivated, with all others remaining completely quiescent (Figure 4B). We noticed a bias toward the reactivation of lateral neuroblasts (Figure 4B′ and data not shown), which may reflect differences in the levels of pathway activation or possibly an intrinsic difference in neuroblast sensitivity to PI3K activity. Normally, the lateral neuroblasts of the thoracic VNC reactivate first (Truman and Bate, 1988), which supports the idea of differential neuroblast sensitivity to dInR/PI3K signaling.

Akt Is Upregulated by PI3K in Neuroblasts and Is Sufficient for Reactivation

Drosophila Akt is a key transducer of increased PIP3 levels, such as those seen in response to dInR/PI3K activation (Oldham et al., 2002; Stocker et al., 2002). Following recruitment to the cell membrane, Akt is activated by PDK1-mediated phosphorylation (Cho et al., 2001; Rintelen et al., 2001). We found that, when we increased PI3K activity in neuroblasts by expression of dp110^{CAAX}, the levels of phosphorylated Akt (pAkt) were concomitantly increased (Figures 4F–F″). To test whether Akt activation is sufficient for the exit from quiescence, we expressed a membrane-targeted form of Akt (myr-Akt) (Stocker et al., 2002) in neuroblasts of larvae reared on a sucrose-only diet. myr-Akt expression was sufficient to drive both growth and cell-cycle re-entry (as evidenced by extensive pH3 labeling) in quiescent neuroblasts in the absence of the nutritional stimulus (Figures 4G–4I and Figure S4). Indeed, expression of myr-Akt was more potent than dp110^{CAAX}, as all grh-GAL4-positive neuroblasts reactivated. The difference in the number of neuroblasts that reactivated in response to dp110^{CAAX} (4%–12%) and myr-Akt (100%) may reflect a differential sensitivity to negative feedback regulation in the pathway (see, for example, Kockel et al., 2010). Myr-Akt may escape negative control more readily than wild-type Akt that has been activated by dp110^{CAAX}.

Once neuroblast reactivation has been ectopically triggered by either PI3K or Akt, then neuroblast proliferation occurs at approximately the same rate. When we assayed reactivated neuroblasts at 24 hr, they had generated on average six or seven daughter cells under either condition. For dp110^{CAAX}, we counted the daughter cells of 29 reactivated neuroblasts from 10 tVNCs; on average, each neuroblast had 6.76 daughter cells. For myr-Akt, we counted the daughter cells of 40 reactivated neuroblasts from four tVNCs; on average, each neuroblast had 6.65 daughter cells. Thus, dInR/PI3K appear to act via their canonical downstream pathway, and when activated in neuroblasts, this pathway is sufficient for reactivation.

dILPs Are Required for Neuroblast Reactivation

There is significant redundancy among the dILP family of InR ligands, with no individual dILP being essential (Grönke et al., 2010). However, two lethal dILP loss-of-function mutant combinations have recently been generated: Δ*dilp 2*,*3*,*5*, and *6*, and Δ*dilp 1*,*2*,*3*,*4*,*5*, and *6* (Grönke et al., 2010). We assayed neuroblast reactivation in the Δ*dilp 2*,*3*,*5*,*6* quadruple mutant. We found no sign of neuroblast reactivation in homozygous *dilp 2*,*3*,*5*,*6* mutants at 28 hr posthatching (compare Figures 5A and 5B). These mutants are developmentally delayed, which could explain the smaller neuroblast size. Therefore, we examined neuroblasts from third-instar mutant larvae that had undergone significant organismal growth. We found that neuroblasts were significantly reduced in size, with many neuroblasts showing no sign of reactivation (Figure 5C). This result is consistent with an acute requirement for dILPs and the insulin/PI3K pathway for neuroblast growth and proliferation.

Glial dILP Expression Is Nutrition Dependent

Are surface glia the source of dILPs that activate dInR/PI3K signaling in neuroblasts in response to nutrition? If so, then we would expect glial dILP expression, or secretion, to be nutrition dependent. It has been demonstrated that nutrition, via the fat body, can control both the expression and secretion of dILPs in the IPC neurons of the brain (Géminard et al., 2009; Ikeya et al., 2002).

When larvae are reared on a sucrose-only diet, there is a significant decrease in surface-glial dILP2 protein expression (compare Figures 5D and 5E). This suggests that glial dILP2 is nutritionally regulated and that this regulation occurs at the level of expression. No antibody is available for dILP6; therefore, we assayed its response to nutrition at the transcript level. We carried out a Q-PCR analysis on the ventral nerve cords from larvae at different developmental times, reared under different nutritional conditions (Figure 5F). We found that the levels of *dilp6* transcript begin to increase by 12 hph and that, by 24 hph, they have increased 8-fold over the levels seen in VNCs from just-hatched larvae (in which neuroblasts are quiescent). Furthermore, the increase in *dilp6* transcription during the first-larval instar is completely abolished when larvae are deprived of amino acids and reared on a sucrose-only diet. Thus, dILP2 and dILP6 expression are both nutrition dependent.

The Glial Expression of dILPs Is Sufficient for Neuroblast Reactivation

If paracrine insulin/IGF signaling from glial cells to neuroblasts is responsible for the nutrition-dependent exit from quiescence, then the forced expression of dILPs within glia should drive neuroblast reactivation in the absence of the systemic nutritional cue. To test this hypothesis, we drove expression of dILP6 (Ikeya et al., 2002) with the glial-specific driver repo-GAL4 (Sepp et al., 2001). When these flies were reared on a sucrose-only diet as

(G and G′) Neuroblasts in which Akt signaling is activated by myr-Akt are quiescent at 0–1 hph.
(H and H′) These neuroblasts can fully reactivate during the first larval instar despite the absence of a nutritional stimulus. The yellow arrow points to a neuroblast not expressing grh-GAL4 that has failed to reactivate in the absence of the nutritional stimulus.
(I and I′) Neuroblasts and their progeny are seen dividing at 48 hph (pH3, white).
See also Figure S4.

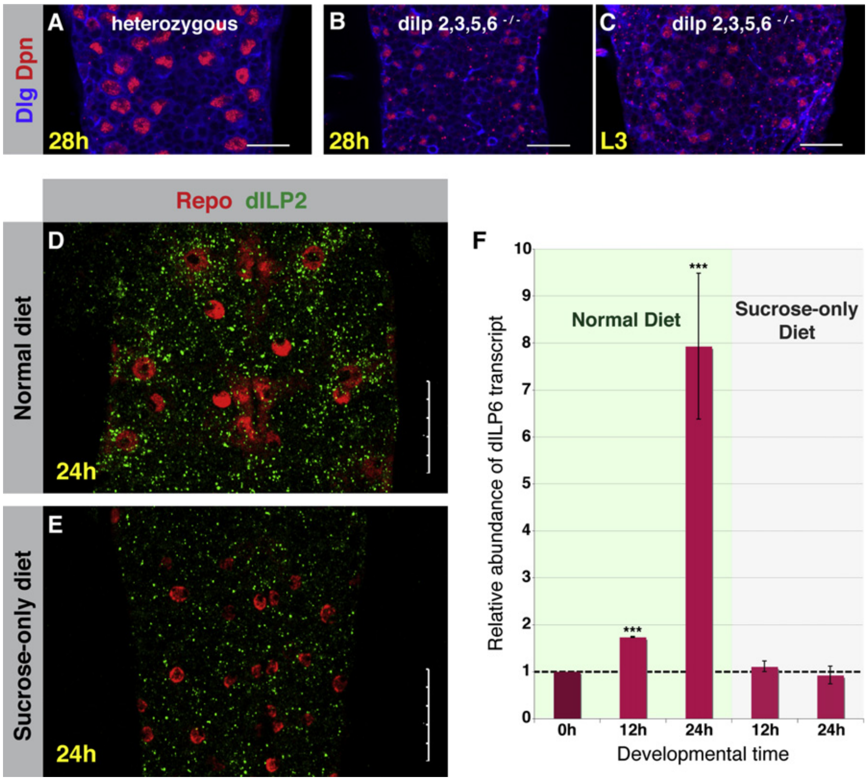

Figure 5. dILPs Are Required for Neuroblast Reactivation, and Their Glial Expression is Nutrition Dependent

(A–C) *dilp 2,3,5,6* mutants display impaired neuroblast reactivation (compare B and C with heterozygous control A). Dpn, red; Dlg, blue. Scale bars, 20 μm.

(D and E) VNCs from Oregon R larvae at 24 hph.

(D) dILP2 protein expression in the surface glia of larvae fed a normal diet.

(E) In larvae reared on a sucrose-only diet, dILP2 expression is greatly reduced (DILP2, green; repo, red). VNCs were dissected, stained, and imaged together. Identical reagents and microscope settings were employed. Scale bars, 25 μm.

(F) Q-PCR analysis of *dilp6* in the VNC. dILP6 transcript levels at 12 hr and 24 hr posthatching in VNCs of larvae fed normal or sucrose-only diets, compared to *dilp6* transcript levels at 0 hr (just hatched). *dilp6* levels normally increase 8-fold during the first instar (0–24 h) but are abolished when larvae are reared on a sucrose-only diet. ***$p < 0.02$; Student's t test. Error bars represent standard deviations. Larvae fed a normal diet showed a mean fold change in *dilp6* mRNA level of 1.7 and 7.9 at 12 and 24 hr, respectively, with SD of 0.01 and 1.55, respectively. Larvae fed a sucrose-only diet showed a mean fold change in *dilp6* mRNA level of 1.1 and 1.2 at 12 and 24 hr, respectively, with SD of 0.11 and 0.15, respectively.

larvae, they initiated neuroblast reactivation despite the absence of organismal growth (Figures 6A and 6B). The enlargement of neuroblasts proceeded as normal, although the reactivated neuroblasts divided less frequently than in fed larvae, with up to four mitotic neuroblasts per VNC at each time point (Figures 6B–6D; n = 17 tVNCs). It may be that maximal pathway activation requires the simultaneous expression of another nutritionally controlled mitogen or that the glial secretion of dILP6 itself is nutritionally regulated.

It has previously been reported that high-level misexpression of dILP2 causes lethality (Ikeya et al., 2002). We found that misexpression of dILP2 using repo-GAL4 caused lethality early in the first-larval instar. We therefore employed the temperature-sensitive GAL4 inhibitor GAL80^{ts} (McGuire et al., 2003) to block expression during embryogenesis. Glial dILP2 expression at larval stages also induced neuroblast reactivation in the absence of amino acids (Figure S5). Taken together, these data support a model in which the nutritional stimulus, acting via the fat body, induces the expression and/or secretion of dILPs by surface glia. These dILPs then act on neuroblasts in a paracrine manner to bring about the growth and proliferation that constitute reactivation (Figure 7E).

Disrupting Glial Signaling Blocks Neuroblast Reactivation

The dILPS are able to substitute for one another functionally (Broughton et al., 2008; Grönke et al., 2010). Consequently, we see no phenotype when we knock down either dILP2 or dILP6 expression in glia by targeted RNAi (data not shown). Furthermore, it has been reported that knockdown of dILP2 expression results in a compensatory increase in transcription of at least two other *dilps* (*dipl3* and *dilp5*) (Broughton et al., 2008; Grönke et al., 2010). To show that glial-derived dILPs are the specific trigger for neuroblast reactivation would require the directed knockdown of at least four dILPs (2, 3, 5, and 6), and possibly more, within glia. To date, such an experiment has not proven technically feasible.

We reasoned that, if glia are the source of dILPs required for neuroblast reactivation, then blocking the ability of glia to signal should inhibit reactivation. To do this, we expressed a dominant-negative, temperature-sensitive mutant of *Drosophila* dynamin (shibire^{ts}; UAS-shi^{ts}) in glial cells to block vesicular trafficking. When we drove expression of shi^{ts} with the glial-specific driver Repo GAL4, we found that neuroblast reactivation was blocked at the restrictive temperature (Figures 7A–7D). Neuroblast growth and proliferation were both dramatically reduced. The block in growth was restricted to neuroblasts; overall regulation of growth was unaffected, and larvae exhibited normal organismal growth and progression through larval stages/instars. We conclude that signaling from the overlying glial cells is crucial for neuroblast reactivation as, importantly, neuroblasts were not reactivated by dILPs secreted from another source. This result supports our model that insulinergic glia are the key relay between nutritional state and neural stem cell reactivation and proliferation (Figure 7E).

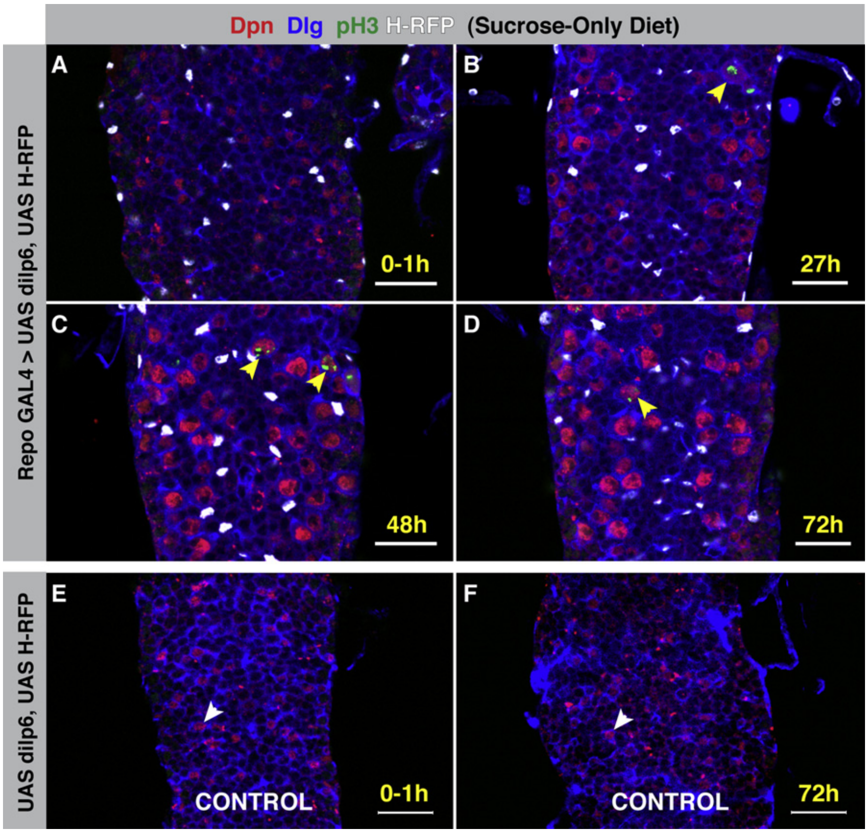

Figure 6. Glial dILP Expression Is Sufficient for Neuroblast Reactivation

(A–D) Repo-GAL4 driving UAS-dilp6 and UAS-Histone H2B-mRFP (H-RFP, white) in larvae reared on a sucrose-only (amino acid-free) diet. Dpn, red; Dlg, blue; pH3, green. Scale bars, 20 μm.

(A) At 0–1 hph, neuroblasts are quiescent, showing no sign of growth or division.

(B) Forced expression of DILP6 in glia drives the reactivation of neuroblasts in the absence of the nutritional stimulus at 27 hr. Yellow arrowheads indicate mitotic neuroblasts.

(C and D) Neuroblasts continue to divide at 48 and 72 hph, respectively. Yellow arrowheads indicate mitotic neuroblasts.

(E and F) Control VNCs from larvae with UAS-dilp6 and UAS-H-RFP, but no GAL4 driver, reared on a sucrose-only (amino acid-free) diet. Neuroblasts never enlarge or divide. White arrowheads indicate neuroblasts. Scale bars, 20 μm.

See also Figure S5.

DISCUSSION

Neuroblast Quiescence and Reactivation

Neuroblast entry into quiescence is governed intrinsically by the same transcription factor cascade that controls neuroblast temporal identity (Isshiki et al., 2001; Tsuji et al., 2008). However, the exit from quiescence and the larval reinitiation of the intrinsic temporal cascade (Maurange et al., 2008) is subject to extrinsic, humoral regulation. It has been reported that, in response to dietary amino acids, the fat body secretes a growth factor/mitogen (FBDM) that acts on the CNS to bring about the cellular growth and cell-cycle re-entry that constitute neuroblast reactivation (Britton and Edgar, 1998). Here, we have identified a population of surface glial cells that respond to the nutrition-dependent stimulus by expressing dILPs and have shown that the dInR/PI3K pathway is required by neuroblasts to exit quiescence in response to nutrition. Forced expression of dILPs in glia or activation of PI3K/Akt signaling in neuroblasts can drive neuroblast growth and proliferation in the absence of dietary protein and thus uncouple neuroblast reactivation from systemic nutritional control.

Cell growth and division are not strictly coupled in neuroblasts. In *Drosophila* Perlecan (dPerlecan) loss-of-function mutants, the majority of neuroblasts appear to increase in size but then remain G1 arrested (Datta, 1995). This suggested that a dedicated mitogen might exist to promote cell-cycle progression. *Drosophila* Activin-like peptides (ALPs) are required for normal levels of neuroblast division in the larval brain and appear to be one such dedicated mitogen (Zhu et al., 2008).

dPerlecan is expressed by glia and forms part of the basement membrane that enwraps the CNS (Friedrich et al., 2000; Lindner et al., 2007; Voigt et al., 2002). dPerlecan was proposed to modulate *Drosophila* FGF (Branchless (Bnl)), allowing it to act as a mitogen for neuroblasts (Park et al., 2003). However, it now appears that the action of Bnl is indirect via a still to be identified cell type (Barrett et al., 2008). One possibility is that Bnl acts on glia to modulate the expression of other proteins, such as dILPs or ALPs, which then in turn act on neuroblasts directly. Here, we show that expression of dILPs by glia leads to neuroblast reactivation in the absence of dietary protein; however, the number of mitoses falls short of that seen under normal dietary conditions. This could be explained by the absence of another nutritionally dependent mitogen. It will be of interest to see whether the glial expression of ALPs, like that of dILPs, relies on dietary protein.

Glia and Neural Stem Cell Proliferation

In the larval CNS, neuroblasts and their progeny are completely surrounded by glial cell processes. If the interaction between neuroblasts and surrounding glia is disrupted by expression of a dominant-negative form of DE-cadherin, the mitotic activity of neuroblasts is severely reduced (Dumstrei et al., 2003). In the mammalian brain, glial cells are involved in a wide variety of processes, including axon guidance, synapse formation, and neuronal specification (reviewed in Ma et al., 2005). Glial cells, with the extracellular matrix and vasculature, also make up the adult neural stem cell niche (reviewed in Nern and Momma, 2006). Astrocytes have been shown to promote neural stem cell

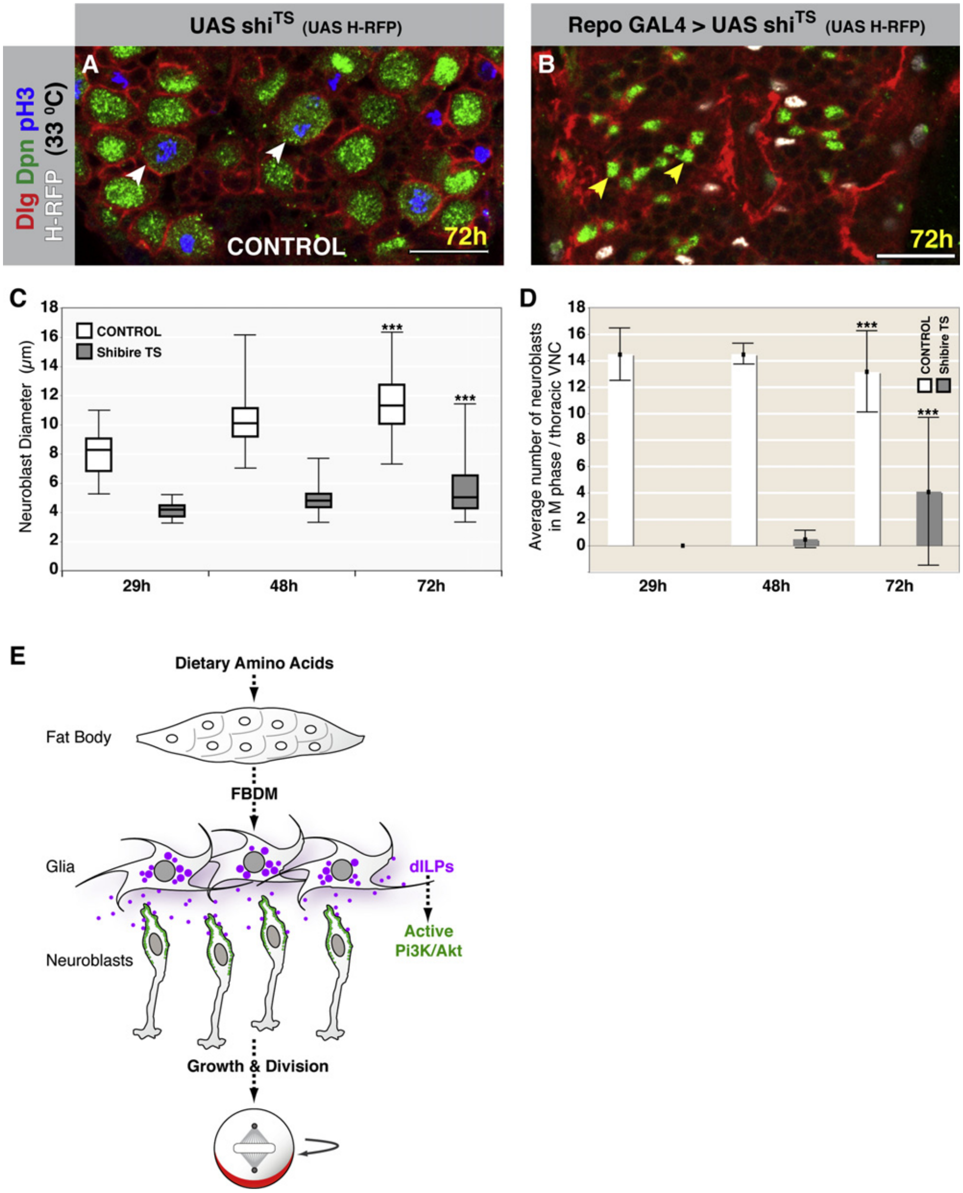

Figure 7. Glia Are a Key Relay between Nutrition and Neuroblast Reactivation

(A and B) Repo-GAL4 driving UAS-shi^{ts} and UAS-Histone H2B-mRFP (H-RFP) and control (no GAL4), reared at 33°C after larval hatching. Dpn, green; Dlg, red; pH3, blue; H-RFP, white. Scale bars, 20 μm.

(A) At 72 hr (midthird instar), neuroblasts in the control are fully enlarged and proliferating. White arrowheads indicate mitotic neuroblasts.

(B) At 72 hr, neuroblasts from animals in which glial dynamin function has been blocked remain quiescent. Yellow arrowheads indicate neuroblasts.

(C and D) Quantification of neuroblast enlargement and proliferation, respectively. ***$p < 0.005$; Student's t test. The higher variation seen at 72 hr posthatching is due to a subset of larvae eventually showing neuroblast reactivation after a prolonged delay (40%; n = 10).

(C) Box and whisker plot showing neuroblast growth is blocked by glial expression of shi^{ts}. At 29, 48, and 72 hr, control neuroblasts have mean diameters of 8.13, 10.27, and 11.46 μm, respectively, with SD of 1.49, 1.71, and 2.06, respectively. At 29, 48, and 72 hr, in larvae in which dynamin function has been blocked in glia, neuroblasts have mean diameters of 4.17, 4.87, and 5.58 μm, respectively, with SD of 0.47, 0.79, and 1.75, respectively.

(D) Bar chart showing neuroblast proliferation is also suppressed by blocking dynamin function in glia. M phase neuroblasts were identified by the presence of pH3. Error bars represent standard deviations. At 29, 48, and 72 hr, control tVNCs have a mean number of M phase neuroblasts of 14.5, 14.6, and 13.14, respectively, with SD of 1.91, 0.71, and 3.02, respectively. At 29, 48, adn 72 hr, in larvae in which dynamin function has been blocked in glia, tVNCs have a mean number of M phase neuroblasts of 0, 0.5, and 4.1, respectively, with SD of 0, 0.58, and 5.55, respectively.

(E) A model for the nutritional control of neuroblast reactivation. Previous work (Britton and Edgar, 1998) suggested that dietary amino acids are sensed by the fat body, triggering FBDM secretion into the hemolymph. The FBDM might then stimulate surface glia, which we show express and secrete dILPs in response to amino acids. These dILPs act on neuroblasts in a paracrine manner to activate the dInR/PI3K/Akt pathway, leading to cell growth and cell-cycle re-entry. dILPs, purple; active PI3K/Akt, green; asymmetrically localized cell fate determinants, red.

proliferation in culture (Song et al., 2002) and can express proproliferative factors such as FGF-2 and IGF-I (Garcia-Estrada et al., 1992; Shetty et al., 2005). Thus, astrocytes are thought to be a key component of the niches that dynamically regulate neural stem cell proliferation in the adult brain (Ma et al., 2005).

We have shown that *Drosophila* surface glia can transduce systemic signals and, by expressing dILP2 and dILP6, control neuroblast exit from quiescence. Glial cells also express dPerlecan and *ana* (Ebens et al., 1993) and are the source of the Activin-like peptides that have been shown to have a direct mitogenic effect on neuroblasts (Brummel et al., 1999; Zhu et al., 2008). Thus, much like mammalian glial cells, *Drosophila* glial cells perform a number of the functions that define a niche and control the proliferation of neural stem cells (Morrison and Spradling, 2008).

Insulin/IGF Signaling and Neural Stem Cell Proliferation

Recent results suggest a role for IGF-1 in the control of neural stem cell division (Mairet-Coello et al., 2009). IGF-1 injection into rat embryonic brain results in a 28% increase in DNA content postnatally as a consequence of increased DNA synthesis and entry into S phase. Conversely, DNA synthesis and entry into S phase are decreased when the PI3K/Akt pathway is blocked. Furthermore, the loss of PTEN, the tumor suppressor and PI3K antagonist, enhances the exit from G_0 of neural stem cells cultured from mouse embryonic cortex (Groszer et al., 2006). The authors suggest that a concomitant increase in cell size may push the cells to enter G_1.

Here, we show, in vivo, that glial expression of insulin-like peptides activates the dInR/PI3K/Akt pathway in *Drosophila* neural stem cells and is responsible for their exit from quiescence. This pathway promotes cell growth and the transition from G_0 to G_1 and is also sufficient to promote G_1-S and mitosis. Given that IGF-1 and the PI3K/Akt pathway can promote cell-cycle progression in vertebrate neural stem cells (Aberg et al., 2003; Yan et al., 2006), this same pathway may regulate vertebrate neural stem cell reactivation in the same way as we have shown here for *Drosophila*.

Manipulating Glia to Control Neuroblast Behavior

The identity of the proposed FBDM, secreted by the fat body in response to dietary protein, remains unknown. However, explant CNS culture experiments demonstrated that the FBDM can act directly on the CNS to bring about neuroblast reactivation (Britton and Edgar, 1998). We have identified the surface glia as a key relay in the nutritional control of neuroblast proliferation. If we can identify the receptor protein(s) that controls glial dILP expression/secretion, then we may, by extension, identify the FBDM and approach a comprehensive understanding of how neural stem cell proliferation is coupled to nutrition and organism-wide growth.

Finding treatments that stimulate the survival and proliferation of endogenous neural stem cells as potential therapies for neurodegenerative disorders is an area of active research (e.g., Androutsellis-Theotokis et al., 2008). The results reported here highlight the effectiveness of targeting support (or niche) cells in order to manipulate the behavior of stem/progenitor cells as an alternative to the direct targeting of the progenitors themselves.

EXPERIMENTAL PROCEDURES

Transgenics

Generation of grh-GAL4: The "D4" *grainyhead* enhancer (~4 kb from the second intron of the *grainyhead* gene) (gift from S. Bray) was excised from pBluescript and ligated into the pPTGAL GAL4 P element vector (Sharma et al., 2002). Generation of dilp6-GAL4: 2 kb, 18 bp "upstream" of the first protein-coding exon of the *dilp6* gene, was amplified from genomic DNA using the PCR primers: forward, GGAATACGAGATACTCCGAAGAAA; reverse, GTTAGATTGCTTAACAACGCTCTG. The resultant PCR product was initially TOPO cloned (Invitrogen), followed by insertion into the pPTGAL GAL4 P element vector. Standard methods were subsequently used for germline transformation.

Quantitative Real-Time PCR

Total RNA was extracted from 60 VNCs (brain dissected away) per sample using TRIZOL reagent (Invitrogen). cDNA was prepared using Superscript II (Invitrogen). Quantitative real-time PCR (Q-PCR) was performed using an ABI 7300 Q-PCR machine and SYBR green (QIAGEN). Results were calculated using the standard curve method and normalized against GAPDH1. Three biological replicates per sample type were generated and each subjected to three technical replicate reactions. dILP6 primers were as in Grönke et al. (2010). GAPDH1 primers used were: forward, ATTTCGCTGAACGATAAGTTCGT; reverse, CGATGACGCGGTTGGAGTA.

Larval Culture

Embryos were placed on a fresh apple juice plate prior to larval hatching. Larvae that hatched within a 30 min window were then transferred to fresh yeast, and this was called 0 hr posthatching (hph). To deprive larvae of dietary amino acids, larvae were transferred to a solution of 20% sucrose in PBS after hatching instead of fresh yeast.

SUPPLEMENTAL INFORMATION

Supplemental Information includes Extended Experimental Procedures and five figures and can be found with this article online at doi:10.1016/j.cell.2010.12.007.

ACKNOWLEDGMENTS

We thank H. Stocker, E. Hafen, S. Bray, S. Leevers, T. Xu, E. Rulifson, C. Doe, J. Skeath, S. Baumgartner, Y. Bellaiche, S. Grönke, L. Partridge, L. Cooley, I. Miguel-Aliaga, the DSHB, and the Bloomington Drosophila Stock Center for reagents. We thank K. Edoff for assistance with confocal microscopy, T. Southall for assistance with microinjection, and Pao-Shu Wu for Q-PCR wisdom. This work was funded by a Wellcome Trust four-year PhD studentship to J.M.C. and a Wellcome Trust Programme Grant to A.H.B.

Received: March 5, 2010
Revised: November 17, 2010
Accepted: December 7, 2010
Published: December 23, 2010

REFERENCES

Aberg, M.A., Aberg, N.D., Palmer, T.D., Alborn, A.M., Carlsson-Skwirut, C., Bang, P., Rosengren, L.E., Olsson, T., Gage, F.H., and Eriksson, P.S. (2003). IGF-I has a direct proliferative effect in adult hippocampal progenitor cells. Mol. Cell. Neurosci. *24*, 23–40.

Ahn, S., and Joyner, A.L. (2005). In vivo analysis of quiescent adult neural stem cells responding to Sonic hedgehog. Nature *437*, 894–897.

Alam, S., Sen, A., Behie, L.A., and Kallos, M.S. (2004). Cell cycle kinetics of expanding populations of neural stem and progenitor cells in vitro. Biotechnol. Bioeng. *88*, 332–347.

Anderson, M.F., Aberg, M.A., Nilsson, M., and Eriksson, P.S. (2002). Insulin-like growth factor-I and neurogenesis in the adult mammalian brain. Brain Res. Dev. Brain Res. *134*, 115–122.

Androutsellis-Theotokis, A., Rueger, M.A., Mkhikian, H., Korb, E., and McKay, R.D. (2008). Signaling pathways controlling neural stem cells slow progressive brain disease. Cold Spring Harb. Symp. Quant. Biol. *73*, 403–410.

Barrett, A.L., Krueger, S., and Datta, S. (2008). Branchless and Hedgehog operate in a positive feedback loop to regulate the initiation of neuroblast division in the Drosophila larval brain. Dev. Biol. *317*, 234–245.

Bier, E., Vaessin, H., Younger-Shepherd, S., Jan, L.Y., and Jan, Y.N. (1992). deadpan, an essential pan-neural gene in Drosophila, encodes a helix-loop-helix protein similar to the hairy gene product. Genes Dev. *6*, 2137–2151.

Britton, J.S., and Edgar, B.A. (1998). Environmental control of the cell cycle in Drosophila: nutrition activates mitotic and endoreplicative cells by distinct mechanisms. Development *125*, 2149–2158.

Britton, J.S., Lockwood, W.K., Li, L., Cohen, S.M., and Edgar, B.A. (2002). Drosophila's insulin/PI3-kinase pathway coordinates cellular metabolism with nutritional conditions. Dev. Cell *2*, 239–249.

Brogiolo, W., Stocker, H., Ikeya, T., Rintelen, F., Fernandez, R., and Hafen, E. (2001). An evolutionarily conserved function of the Drosophila insulin receptor and insulin-like peptides in growth control. Curr. Biol. *11*, 213–221.

Broughton, S., Alic, N., Slack, C., Bass, T., Ikeya, T., Vinti, G., Tommasi, A.M., Driege, Y., Hafen, E., and Partridge, L. (2008). Reduction of DILP2 in Drosophila triages a metabolic phenotype from lifespan revealing redundancy and compensation among DILPs. PLoS ONE *3*, e3721.

Brummel, T., Abdollah, S., Haerry, T.E., Shimell, M.J., Merriam, J., Raftery, L., Wrana, J.L., and O'Connor, M.B. (1999). The Drosophila activin receptor baboon signals through dSmad2 and controls cell proliferation but not patterning during larval development. Genes Dev. *13*, 98–111.

Cho, K.S., Lee, J.H., Kim, S., Kim, D., Koh, H., Lee, J., Kim, C., Kim, J., and Chung, J. (2001). Drosophila phosphoinositide-dependent kinase-1 regulates apoptosis and growth via the phosphoinositide 3-kinase-dependent signaling pathway. Proc. Natl. Acad. Sci. USA *98*, 6144–6149.

Coller, H.A., Sang, L., and Roberts, J.M. (2006). A new description of cellular quiescence. PLoS Biol. *4*, e83.

Colombani, J., Raisin, S., Pantalacci, S., Radimerski, T., Montagne, J., and Léopold, P. (2003). A nutrient sensor mechanism controls Drosophila growth. Cell *114*, 739–749.

Datta, S. (1995). Control of proliferation activation in quiescent neuroblasts of the Drosophila central nervous system. Development *121*, 1173–1182.

Doetsch, F., García-Verdugo, J.M., and Alvarez-Buylla, A. (1999). Regeneration of a germinal layer in the adult mammalian brain. Proc. Natl. Acad. Sci. USA *96*, 11619–11624.

Drummond-Barbosa, D. (2008). Stem cells, their niches and the systemic environment: an aging network. Genetics *180*, 1787–1797.

Dumstrei, K., Wang, F., and Hartenstein, V. (2003). Role of DE-cadherin in neuroblast proliferation, neural morphogenesis, and axon tract formation in Drosophila larval brain development. J. Neurosci. *23*, 3325–3335.

Ebens, A.J., Garren, H., Cheyette, B.N., and Zipursky, S.L. (1993). The Drosophila anachronism locus: a glycoprotein secreted by glia inhibits neuroblast proliferation. Cell *74*, 15–27.

Egger, B., Chell, J.M., and Brand, A.H. (2008). Insights into neural stem cell biology from flies. Philos. Trans. R. Soc. Lond. B Biol. Sci. *363*, 39–56.

Fernandez, R., Tabarini, D., Azpiazu, N., Frasch, M., and Schlessinger, J. (1995). The Drosophila insulin receptor homolog: a gene essential for embryonic development encodes two receptor isoforms with different signaling potential. EMBO J. *14*, 3373–3384.

Friedrich, M.V., Schneider, M., Timpl, R., and Baumgartner, S. (2000). Perlecan domain V of Drosophila melanogaster. Sequence, recombinant analysis and tissue expression. Eur. J. Biochem. *267*, 3149–3159.

Garcia-Estrada, J., Garcia-Segura, L.M., and Torres-Aleman, I. (1992). Expression of insulin-like growth factor I by astrocytes in response to injury. Brain Res. *592*, 343–347.

Garofalo, R.S., and Rosen, O.M. (1988). Tissue localization of Drosophila melanogaster insulin receptor transcripts during development. Mol. Cell. Biol. *8*, 1638–1647.

Géminard, C., Rulifson, E.J., and Léopold, P. (2009). Remote control of insulin secretion by fat cells in Drosophila. Cell Metab. *10*, 199–207.

Goberdhan, D.C., and Wilson, C. (2003). The functions of insulin signaling: size isn't everything, even in Drosophila. Differentiation *71*, 375–397.

Goberdhan, D.C., Paricio, N., Goodman, E.C., Mlodzik, M., and Wilson, C. (1999). Drosophila tumor suppressor PTEN controls cell size and number by antagonizing the Chico/PI3-kinase signaling pathway. Genes Dev. *13*, 3244–3258.

Grönke, S., Clarke, D.F., Broughton, S., Andrews, T.D., and Partridge, L. (2010). Molecular evolution and functional characterization of Drosophila insulin-like peptides. PLoS Genet. *6*, e1000857.

Groszer, M., Erickson, R., Scripture-Adams, D.D., Dougherty, J.D., Le Belle, J., Zack, J.A., Geschwind, D.H., Liu, X., Kornblum, H.I., and Wu, H. (2006). PTEN negatively regulates neural stem cell self-renewal by modulating G0-G1 cell cycle entry. Proc. Natl. Acad. Sci. USA *103*, 111–116.

Hartenstein, V., Rudloff, E., and Campos-Ortega, J.A. (1987). The pattern of proliferation of the neuroblasts in the wild-type embryo of *Drosophila melanogaster*. Rouxs Arch. Dev. Biol. *196*, 473–485.

Huang, H., Potter, C.J., Tao, W., Li, D.M., Brogiolo, W., Hafen, E., Sun, H., and Xu, T. (1999). PTEN affects cell size, cell proliferation and apoptosis during Drosophila eye development. Development *126*, 5365–5372.

Ikeya, T., Galic, M., Belawat, P., Nairz, K., and Hafen, E. (2002). Nutrient-dependent expression of insulin-like peptides from neuroendocrine cells in the CNS contributes to growth regulation in Drosophila. Curr. Biol. *12*, 1293–1300.

Isshiki, T., Pearson, B., Holbrook, S., and Doe, C.Q. (2001). Drosophila neuroblasts sequentially express transcription factors which specify the temporal identity of their neuronal progeny. Cell *106*, 511–521.

Ito, K., and Hotta, Y. (1992). Proliferation pattern of postembryonic neuroblasts in the brain of Drosophila melanogaster. Dev. Biol. *149*, 134–148.

Joseph D'Ercole, A., and Ye, P. (2008). Expanding the mind: insulin-like growth factor I and brain development. Endocrinology *149*, 5958–5962.

Knoblich, J.A. (2008). Mechanisms of asymmetric stem cell division. Cell *132*, 583–597.

Kockel, L., Kerr, K.S., Melnick, M., Brückner, K., Hebrok, M., and Perrimon, N. (2010). Dynamic switch of negative feedback regulation in Drosophila Akt-TOR signaling. PLoS Genet. *6*, e1000990.

LaFever, L., and Drummond-Barbosa, D. (2005). Direct control of germline stem cell division and cyst growth by neural insulin in Drosophila. Science *309*, 1071–1073.

Leevers, S.J., Weinkove, D., MacDougall, L.K., Hafen, E., and Waterfield, M.D. (1996). The Drosophila phosphoinositide 3-kinase Dp110 promotes cell growth. EMBO J. *15*, 6584–6594.

Lindner, J.R., Hillman, P.R., Barrett, A.L., Jackson, M.C., Perry, T.L., Park, Y., and Datta, S. (2007). The Drosophila Perlecan gene trol regulates multiple signaling pathways in different developmental contexts. BMC Dev. Biol. *7*, 121.

Lizcano, J.M., Alrubaie, S., Kieloch, A., Deak, M., Leevers, S.J., and Alessi, D.R. (2003). Insulin-induced Drosophila S6 kinase activation requires phosphoinositide 3-kinase and protein kinase B. Biochem. J. *374*, 297–306.

Ma, D.K., Ming, G.L., and Song, H. (2005). Glial influences on neural stem cell development: cellular niches for adult neurogenesis. Curr. Opin. Neurobiol. *15*, 514–520.

Ma, D.K., Bonaguidi, M.A., Ming, G.L., and Song, H. (2009). Adult neural stem cells in the mammalian central nervous system. Cell Res. *19*, 672–682.

Maehama, T., and Dixon, J.E. (1998). The tumor suppressor, PTEN/MMAC1, dephosphorylates the lipid second messenger, phosphatidylinositol 3,4,5-trisphosphate. J. Biol. Chem. *273*, 13375–13378.

Mairet-Coello, G., Tury, A., and DiCicco-Bloom, E. (2009). Insulin-like growth factor-1 promotes G(1)/S cell cycle progression through bidirectional regulation of cyclins and cyclin-dependent kinase inhibitors via the phosphatidylinositol 3-kinase/Akt pathway in developing rat cerebral cortex. J. Neurosci. *29*, 775–788.

Maurange, C., Cheng, L., and Gould, A.P. (2008). Temporal transcription factors and their targets schedule the end of neural proliferation in Drosophila. Cell *133*, 891–902.

McGuire, S.E., Le, P.T., Osborn, A.J., Matsumoto, K., and Davis, R.L. (2003). Spatiotemporal rescue of memory dysfunction in Drosophila. Science *302*, 1765–1768.

Miron, M., Lasko, P., and Sonenberg, N. (2003). Signaling from Akt to FRAP/TOR targets both 4E-BP and S6K in Drosophila melanogaster. Mol. Cell. Biol. *23*, 9117–9126.

Moore, K.A., and Lemischka, I.R. (2006). Stem cells and their niches. Science *311*, 1880–1885.

Morrison, S.J., and Spradling, A.C. (2008). Stem cells and niches: mechanisms that promote stem cell maintenance throughout life. Cell *132*, 598–611.

Morshead, C.M., Reynolds, B.A., Craig, C.G., McBurney, M.W., Staines, W.A., Morassutti, D., Weiss, S., and van der Kooy, D. (1994). Neural stem cells in the adult mammalian forebrain: a relatively quiescent subpopulation of subependymal cells. Neuron *13*, 1071–1082.

Nern, C., and Momma, S. (2006). The realized niche of adult neural stem cells. Stem Cell Rev. *2*, 233–240.

Okamoto, N., Yamanaka, N., Yagi, Y., Nishida, Y., Kataoka, H., O'Connor, M.B., and Mizoguchi, A. (2009). A fat body-derived IGF-like peptide regulates postfeeding growth in Drosophila. Dev. Cell *17*, 885–891.

Oldham, S., Stocker, H., Laffargue, M., Wittwer, F., Wymann, M., and Hafen, E. (2002). The Drosophila insulin/IGF receptor controls growth and size by modulating PtdInsP(3) levels. Development *129*, 4103–4109.

Park, Y., Rangel, C., Reynolds, M.M., Caldwell, M.C., Johns, M., Nayak, M., Welsh, C.J., McDermott, S., and Datta, S. (2003). Drosophila perlecan modulates FGF and hedgehog signals to activate neural stem cell division. Dev. Biol. *253*, 247–257.

Prokop, A., Bray, S., Harrison, E., and Technau, G.M. (1998). Homeotic regulation of segment-specific differences in neuroblast numbers and proliferation in the Drosophila central nervous system. Mech. Dev. *74*, 99–110.

Prokop, A., and Technau, G.M. (1991). The origin of postembryonic neuroblasts in the ventral nerve cord of Drosophila melanogaster. Development *111*, 79–88.

Rintelen, F., Stocker, H., Thomas, G., and Hafen, E. (2001). PDK1 regulates growth through Akt and S6K in Drosophila. Proc. Natl. Acad. Sci. USA *98*, 15020–15025.

Rulifson, E.J., Kim, S.K., and Nusse, R. (2002). Ablation of insulin-producing neurons in flies: growth and diabetic phenotypes. Science *296*, 1118–1120.

Sepp, K.J., Schulte, J., and Auld, V.J. (2001). Peripheral glia direct axon guidance across the CNS/PNS transition zone. Dev. Biol. *238*, 47–63.

Sharma, Y., Cheung, U., Larsen, E.W., and Eberl, D.F. (2002). PPTGAL, a convenient Gal4 P-element vector for testing expression of enhancer fragments in drosophila. Genesis *34*, 115–118.

Shetty, A.K., Hattiangady, B., and Shetty, G.A. (2005). Stem/progenitor cell proliferation factors FGF-2, IGF-1, and VEGF exhibit early decline during the course of aging in the hippocampus: role of astrocytes. Glia *51*, 173–186.

Slaidina, M., Delanoue, R., Gronke, S., Partridge, L., and Léopold, P. (2009). A Drosophila insulin-like peptide promotes growth during nonfeeding states. Dev. Cell *17*, 874–884.

Song, H., Stevens, C.F., and Gage, F.H. (2002). Astroglia induce neurogenesis from adult neural stem cells. Nature *417*, 39–44.

Stocker, H., Andjelkovic, M., Oldham, S., Laffargue, M., Wymann, M.P., Hemmings, B.A., and Hafen, E. (2002). Living with lethal PIP3 levels: viability of flies lacking PTEN restored by a PH domain mutation in Akt/PKB. Science *295*, 2088–2091.

Stork, T., Engelen, D., Krudewig, A., Silies, M., Bainton, R.J., and Klämbt, C. (2008). Organization and function of the blood-brain barrier in Drosophila. J. Neurosci. *28*, 587–597.

Truman, J.W., and Bate, M. (1988). Spatial and temporal patterns of neurogenesis in the central nervous system of Drosophila melanogaster. Dev. Biol. *125*, 145–157.

Tsuji, T., Hasegawa, E., and Isshiki, T. (2008). Neuroblast entry into quiescence is regulated intrinsically by the combined action of spatial Hox proteins and temporal identity factors. Development *135*, 3859–3869.

Uv, A.E., Harrison, E.J., and Bray, S.J. (1997). Tissue-specific splicing and functions of the Drosophila transcription factor Grainyhead. Mol. Cell. Biol. *17*, 6727–6735.

Verdu, J., Buratovich, M.A., Wilder, E.L., and Birnbaum, M.J. (1999). Cell-autonomous regulation of cell and organ growth in Drosophila by Akt/PKB. Nat. Cell Biol. *1*, 500–506.

Voigt, A., Pflanz, R., Schäfer, U., and Jäckle, H. (2002). Perlecan participates in proliferation activation of quiescent Drosophila neuroblasts. Dev. Dyn. *224*, 403–412.

Weinkove, D., Neufeld, T.P., Twardzik, T., Waterfield, M.D., and Leevers, S.J. (1999). Regulation of imaginal disc cell size, cell number and organ size by Drosophila class I(A) phosphoinositide 3-kinase and its adaptor. Curr. Biol. *9*, 1019–1029.

Woodward, W.A., Chen, M.S., Behbod, F., and Rosen, J.M. (2005). On mammary stem cells. J. Cell Sci. *118*, 3585–3594.

Yan, Y.P., Sailor, K.A., Vemuganti, R., and Dempsey, R.J. (2006). Insulin-like growth factor-1 is an endogenous mediator of focal ischemia-induced neural progenitor proliferation. Eur. J. Neurosci. *24*, 45–54.

Ye, P., Popken, G.J., Kemper, A., McCarthy, K., Popko, B., and D'Ercole, A.J. (2004). Astrocyte-specific overexpression of insulin-like growth factor-I promotes brain overgrowth and glial fibrillary acidic protein expression. J. Neurosci. Res. *78*, 472–484.

Zammit, P.S. (2008). All muscle satellite cells are equal, but are some more equal than others? J. Cell Sci. *121*, 2975–2982.

Zhao, C., Deng, W., and Gage, F.H. (2008). Mechanisms and functional implications of adult neurogenesis. Cell *132*, 645–660.

Zhu, C.C., Boone, J.Q., Jensen, P.A., Hanna, S., Podemski, L., Locke, J., Doe, C.Q., and O'Connor, M.B. (2008). Drosophila Activin- and the Activin-like product Dawdle function redundantly to regulate proliferation in the larval brain. Development *135*, 513–521.

EXTENDED EXPERIMENTAL PROCEDURES

Immunohistochemistry

Larval CNS was dissected in PBS, then fixed for 15–20 min in PBS containing 4% formaldehyde (ultra pure), 0.5mM EGTA, and 5mM $MgCl_2$. Wash solution was PBS with 0.3% Triton X-100. Primary antibodies used were: rabbit anti GFP (1 in 1000) (ab6556, Abcam), chicken anti GFP (1 in 20) (06-896, Upstate), mouse anti GFP (1 in 20) (11814460001, Roche), mouse anti Discs Large (c) (1 in 70) (4F3, Developmental Studies Hybridoma Bank (DSHB)), Rat anti ElaV (c) (1 in 70) (7E8A10, DSHB), Rat anti Deadpan (8 in 10) (C.Q. Doe), Guinea Pig anti Deadpan (1 in 500) (J.B. Skeath), mouse anti Repo (c) (1 in 70) (8D12, DSHB), rabbit anti dILP2 (1 in 400) (E. Rulifson), rabbit anti dPerlecan (1 in 2000) (S. Baumgartner), rabbit anti pH3 (1 in 100) (06-570, Upstate), Guinea Pig anti Miranda (1 in 200) (A.H. Brand), mouse anti Prospero (c) (1 in 70) (MR1A, DSHB), rabbit anti pAkt (1 in 75) (D9E, Cell Signaling Technology). Appropriate combinations of Alexa-coupled secondary antibodies (Invitrogen) were subsequently applied. Samples were analyzed with a Leica SP2, or Zeis LSM510 confocal microscope.

Image Processing

Imaris and Volocity were used to process confocal data. Adobe Photoshop and Illustrator were used to generate figures.

Fly lines

UAS-dilp2 and UAS-dilp6 (Ikeya et al., 2002). UAS-myr-Akt (Stocker et al., 2002). UAS-dp110^{CAAX} (Leevers et al., 1996). UAS-Δp60 (Weinkove et al., 1999). dp110^{A} and dp110^{B} null mutants (Weinkove et al., 1999). UAS-dPTEN (Huang et al., 1999). UAS-Histone H2B-mRFP (Langevin et al., 2005). tub > PH-GFP (Britton et al., 2002). S6K GFP protein-trap (Buszczak et al., 2007). ΔdILP 2,3,5,6 quadruple mutant (Grönke et al., 2010). tub > GAL80^{ts} (McGuire et al., 2003). UAS-mCD8-GFP (on the second or third chromosome) (Lee and Luo, 1999), repo-GAL4 (Sepp et al., 2001), UAS-shi^{ts} (Kitamoto, 2001) UAS-InR^{K1409A} (we combined the insertions on the second and third chromosomes for use in our experiments) (Exelixis, Inc.), and Oregon-R, were acquired from the Bloomington *Drosophila* stock center.

SUPPLEMENTAL REFERENCES

Britton, J.S., Lockwood, W.K., Li, L., Cohen, S.M., and Edgar, B.A. (2002). Drosophila's insulin/PI3-kinase pathway coordinates cellular metabolism with nutritional conditions. Dev. Cell *2*, 239–249.

Buszczak, M., Paterno, S., Lighthouse, D., Bachman, J., Planck, J., Owen, S., Skora, A.D., Nystul, T.G., Ohlstein, B., Allen, A., et al. (2007). The carnegie protein trap library: a versatile tool for Drosophila developmental studies. Genetics *175*, 1505–1531.

Grönke, S., Clarke, D.F., Broughton, S., Andrews, T.D., and Partridge, L. (2010). Molecular evolution and functional characterization of Drosophila insulin-like peptides. PLoS Genet. *6*, e1000857.

Huang, H., Potter, C.J., Tao, W., Li, D.M., Brogiolo, W., Hafen, E., Sun, H., and Xu, T. (1999). PTEN affects cell size, cell proliferation and apoptosis during Drosophila eye development. Development *126*, 5365–5372.

Ikeya, T., Galic, M., Belawat, P., Nairz, K., and Hafen, E. (2002). Nutrient-dependent expression of insulin-like peptides from neuroendocrine cells in the CNS contributes to growth regulation in Drosophila. Curr. Biol. *12*, 1293–1300.

Kelso, R.J., Buszczak, M., Quiñones, A.T., Castiblanco, C., Mazzalupo, S., and Cooley, L. (2004). Flytrap, a database documenting a GFP protein-trap insertion screen in *Drosophila melanogaster.* Nucleic Acids Res. *32* (*Database issue*), D418–D420.

Kitamoto, T. (2001). Conditional modification of behavior in Drosophila by targeted expression of a temperature-sensitive shibire allele in defined neurons. J. Neurobiol. *47*, 81–92.

Langevin, J., Le Borgne, R., Rosenfeld, F., Gho, M., Schweisguth, F., and Bellaïche, Y. (2005). Lethal giant larvae controls the localization of notch-signaling regulators numb, neuralized, and Sanpodo in Drosophila sensory-organ precursor cells. Curr. Biol. *15*, 955–962.

Lee, T., and Luo, L. (1999). Mosaic analysis with a repressible cell marker for studies of gene function in neuronal morphogenesis. Neuron *22*, 451–461.

Leevers, S.J., Weinkove, D., MacDougall, L.K., Hafen, E., and Waterfield, M.D. (1996). The Drosophila phosphoinositide 3-kinase Dp110 promotes cell growth. EMBO J. *15*, 6584–6594.

McGuire, S.E., Le, P.T., Osborn, A.J., Matsumoto, K., and Davis, R.L. (2003). Spatiotemporal rescue of memory dysfunction in Drosophila. Science *302*, 1765–1768.

Sepp, K.J., Schulte, J., and Auld, V.J. (2001). Peripheral glia direct axon guidance across the CNS/PNS transition zone. Dev. Biol. *238*, 47–63.

Stocker, H., Andjelkovic, M., Oldham, S., Laffargue, M., Wymann, M.P., Hemmings, B.A., and Hafen, E. (2002). Living with lethal PIP3 levels: viability of flies lacking PTEN restored by a PH domain mutation in Akt/PKB. Science *295*, 2088–2091.

Weinkove, D., Neufeld, T.P., Twardzik, T., Waterfield, M.D., and Leevers, S.J. (1999). Regulation of imaginal disc cell size, cell number and organ size by Drosophila class I(A) phosphoinositide 3-kinase and its adaptor. Curr. Biol. *9*, 1019–1029.

Cell

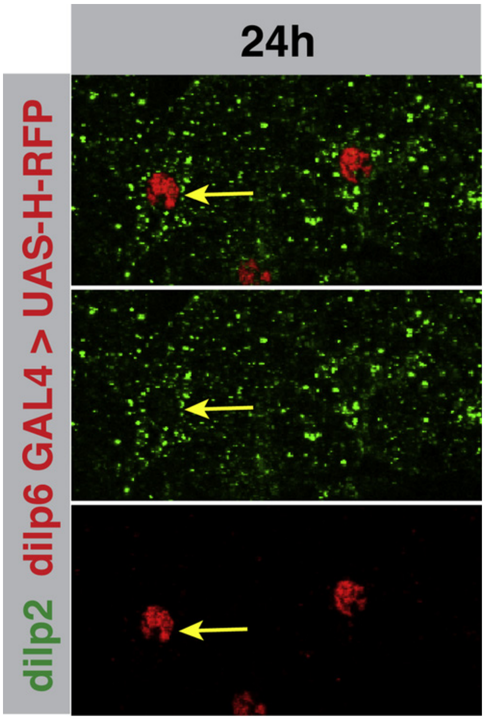

Figure S1. dILP6 and dILP2 Are Expressed in the Same Cells, Related to Figure 2

24h VNC in which dilp6-GAL4 is driving the nuclear marker histone-mRFP (in red) (under UAS control). Glial cells expressing dILP2 protein (in green) are the same cells that express dilp6 GAL4 (red nuclei). Compare yellow arrows between the separate channels and overlay.

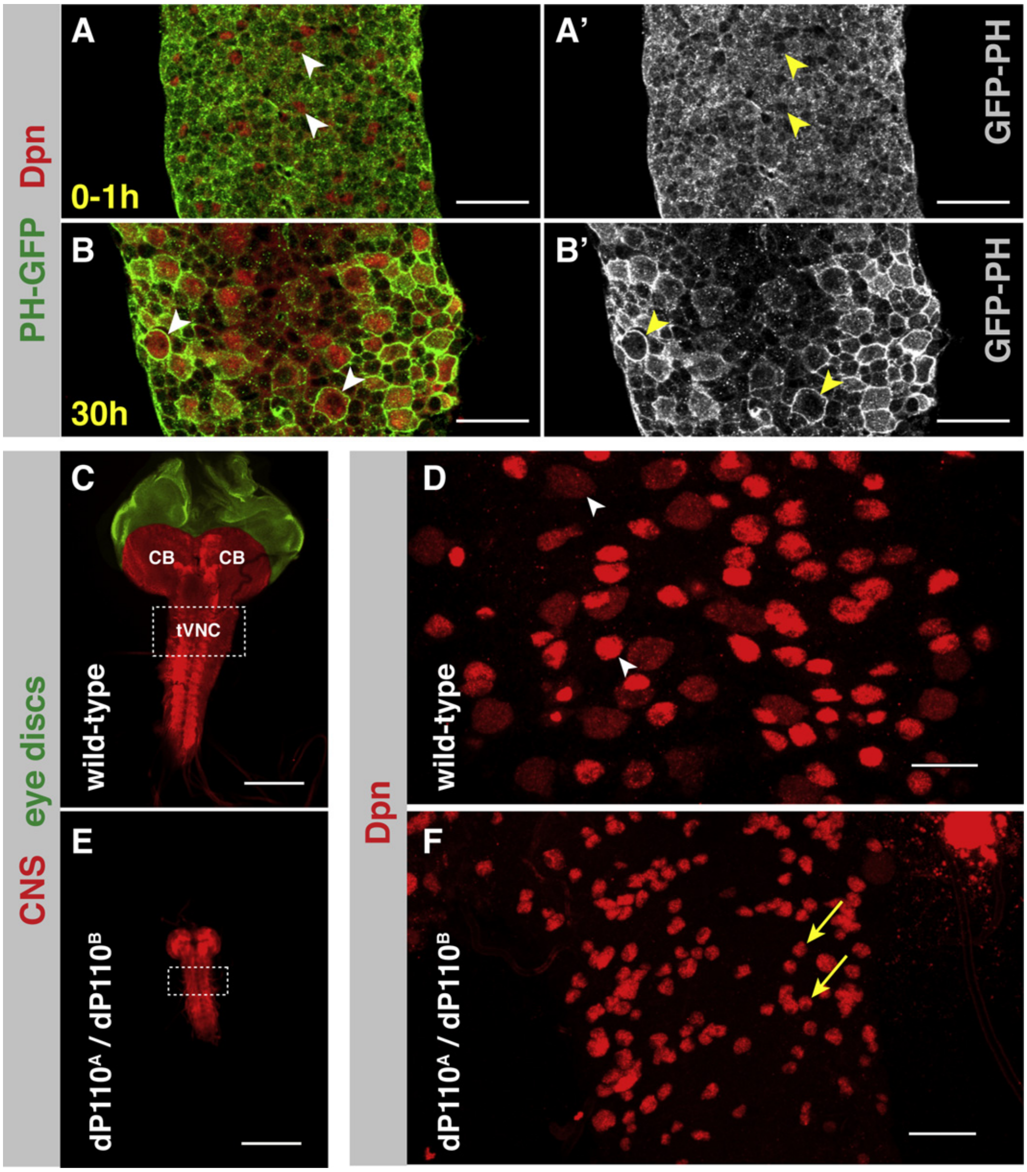

Figure S2. PI3K Is Active during, and Required for, Neuroblast Reactivation, Related to Figure 3

(A–B′) PH-GFP distribution during reactivation. During neuroblast reactivation there is a strong accumulation of PH-GFP at the cell membrane (compare arrowheads in A, A' (quiescent neuroblasts) to those in B,B' (reactivating neuroblasts)), indicating increased PIP3 levels and therefore increased PI3K activity. (GFP in green, Deadpan in red, scale bars represent 20μm).

(C and E) The CNS is significantly reduced in size in PI3K (dp110) loss of function mutants. CNS from wandering third instar larvae stained with Discs large and false colored (CNS in red, and eye discs in green). Thoracic VNC (tVNC) highlighted with dashed white box. CB marks the central brain. (Scale bars represent 150μm).

(D and F) Neuroblasts remain quiescent in PI3K (dp110) mutants. z-projections of the tVNC. In PI3K mutants, neuroblast cell growth is significantly retarded, with many neuroblasts remaining quiescent. Compare yellow arrows in (F), with white arrowheads in (D). (Dpn in red, scale bars represent 20μm).

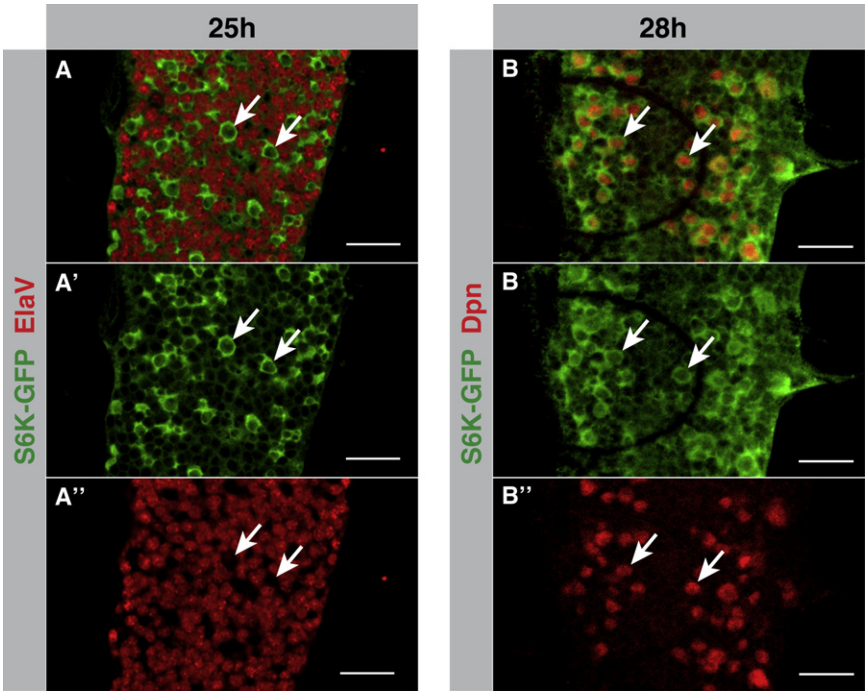

Figure S3. S6K Is Enriched in Reactivating Neuroblasts, Related to Figure 3

Late first instar larval VNCs (genotype exhibits slightly delayed development) that are homozygous for the S6K-GFP protein trap (in which GFP has been inserted into the second intron of endogenous S6K) (Buszczak et al., 2007; Kelso et al., 2004).

(A–A″) During reactivation S6K is enriched in a population of CNS cells which are negative for the neuron-specific transcription factor ElaV (in red) (see white arrows).

(B–B″) The ElaV-negative cells with high S6K GFP levels are the neuroblasts, as evidenced by their Deadpan-positive nuclei (in red) (see white arrows).

(Scale bars in all panels represent 20μm).

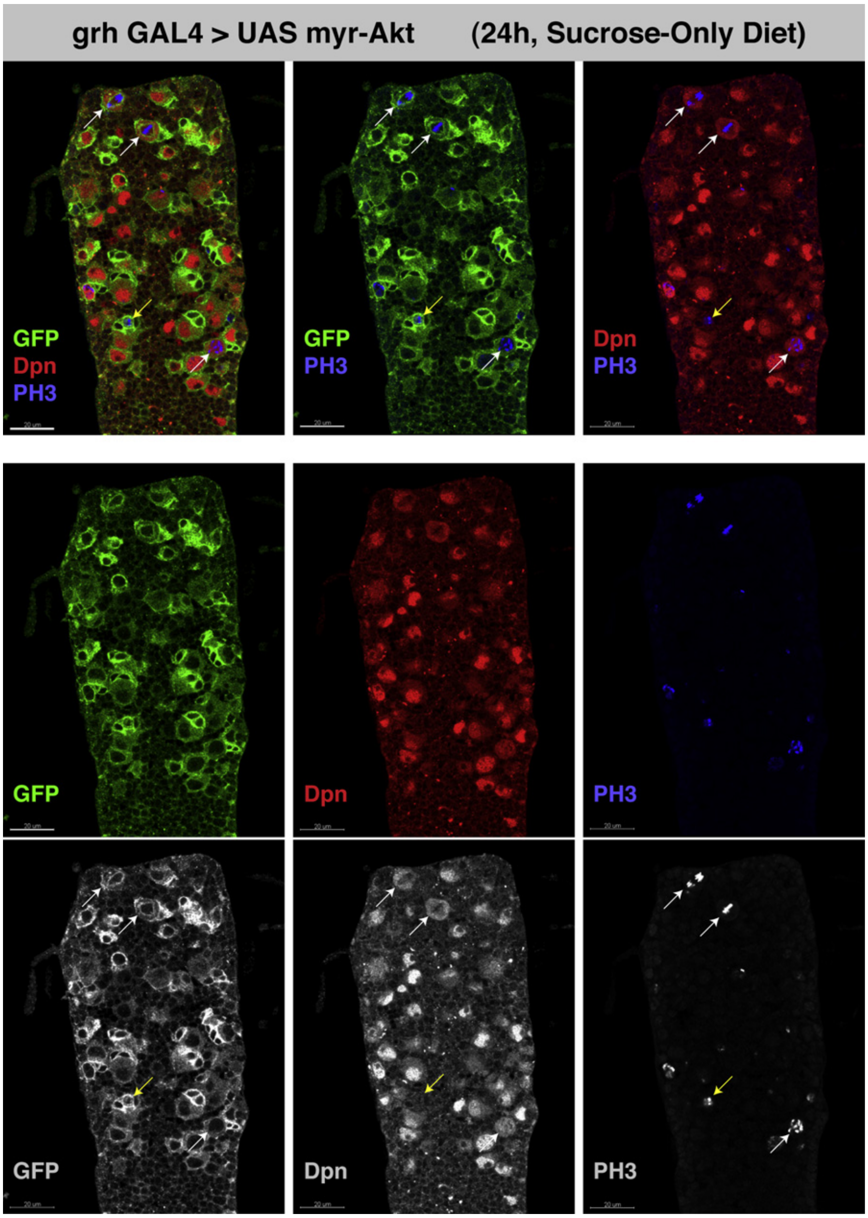

Figure S4. Akt Signaling Is Sufficient for Neuroblast Growth and Cell-Cycle Re-Entry, Related to Figure 4

24h VNC from a larva reared on a sucrose-only diet in which grh-GAL4 is driving UAS myr-Akt and UAS mCD8-GFP expression. myr-Akt expression is sufficient for neuroblast reactivation in the absence of the amino-acid stimulus.

Confocal channels have been split to allow better visualization of representative mitotic figures. All grh-Gal4-positive neuroblasts have enlarged, and many have begun to divide as evidenced by smaller, GFP-retaining, daughter cells adjacent to the neuroblasts, and pH3 staining. Mitotic pH3 nuclei can be seen in both neuroblasts (white arrows), and neuroblast progeny (yellow arrow). (Scale bars represent 20μm).

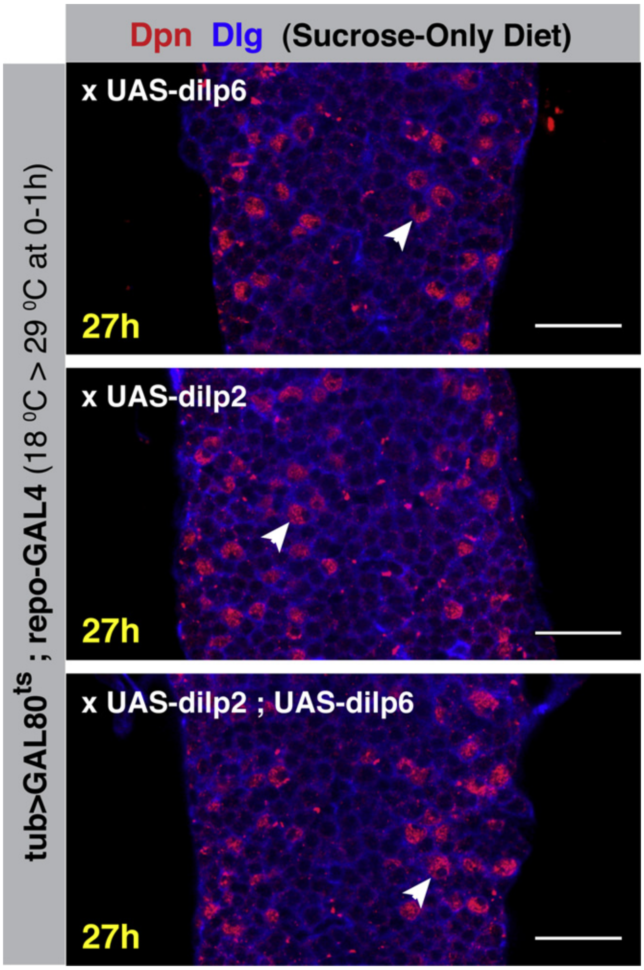

Figure S5. dILP2 Can Also Reactivate Neuroblasts, Related to Figure 6

27h VNCs from larvae reared on a sucrose-only diet in which repo-GAL4 is driving the expression of either dILP6, dILP2, or dILP2 and dILP6, in the presence of tubGAL80^{ts}. Larvae were shifted from 18°C to 29°C at larval hatching to block the repressor activity of GAL80, and allow dilp expression. Both dILP6 and dILP2 were sufficient for neuroblast reactivation under these conditions (white arrowheads point to enlarging neuroblasts). Co-expression of dILP2 and dILP6 had no additive or synergistic effect on neuroblast reactivation under these conditions. (Scale bars represent 20μm).
